# The expression of Rpb10, a small subunit common to RNA polymerases, is modulated by the R3H domain-containing Rbs1 protein and the Upf1 helicase

**DOI:** 10.1093/nar/gkaa1069

**Published:** 2020-11-24

**Authors:** Małgorzata Cieśla, Tomasz W Turowski, Marcin Nowotny, David Tollervey, Magdalena Boguta

**Affiliations:** Department of Genetics, Institute of Biochemistry and Biophysics, Polish Academy of Sciences, Pawińskiego 5A, 02-106 Warsaw, Poland; Wellcome Centre for Cell Biology, The University of Edinburgh, Edinburgh EH9 3BF, Scotland; Laboratory of Protein Structure, International Institute of Molecular and Cell Biology, Ks. Trojdena 4, 02-109 Warsaw, Poland; Wellcome Centre for Cell Biology, The University of Edinburgh, Edinburgh EH9 3BF, Scotland; Department of Genetics, Institute of Biochemistry and Biophysics, Polish Academy of Sciences, Pawińskiego 5A, 02-106 Warsaw, Poland

## Abstract

The biogenesis of eukaryotic RNA polymerases is poorly understood. The present study used a combination of genetic and molecular approaches to explore the assembly of RNA polymerase III (Pol III) in yeast. We identified a regulatory link between Rbs1, a Pol III assembly factor, and Rpb10, a small subunit that is common to three RNA polymerases. Overexpression of Rbs1 increased the abundance of both *RPB10* mRNA and the Rpb10 protein, which correlated with suppression of Pol III assembly defects. Rbs1 is a poly(A)mRNA-binding protein and mutational analysis identified R3H domain to be required for mRNA interactions and genetic enhancement of Pol III biogenesis. Rbs1 also binds to Upf1 protein, a key component in nonsense-mediated mRNA decay (NMD) and levels of *RPB10* mRNA were increased in a *upf1Δ* strain. Genome-wide RNA binding by Rbs1 was characterized by UV cross-linking based approach. We demonstrated that Rbs1 directly binds to the 3′ untranslated regions (3′UTRs) of many mRNAs including transcripts encoding Pol III subunits, Rpb10 and Rpc19. We propose that Rbs1 functions by opposing mRNA degradation, at least in part mediated by NMD pathway. Orthologues of Rbs1 protein are present in other eukaryotes, including humans, suggesting that this is a conserved regulatory mechanism.

## INTRODUCTION

Transcription of the eukaryotic genome requires at least three different multisubunit RNA polymerases. Insights into understanding the assembly of polymerase complexes have been provided by recent findings of their structures in a model eukaryotic organism, *Saccharomyces cerevisiae* (for review, see (1)). The yeast RNA polymerases Pol I, Pol II and Pol III contain 14, 12 and 17 subunits, respectively, and share a 10-subunit catalytic core that consists of identical or related proteins. The active center cleft is formed by the two largest subunits that harbor catalytic activity and are related to the β′ and β components of the α_2_ββ′ core of bacterial RNA polymerase. Homology to the bacterial α subunit, although less strong, was also observed for the Rpc40 subunit, which is common to Pol I and Pol III, and Rpb3, the analogue of Rpc40 in Pol II. This α-like subunit forms a heterodimer with a second α-like subunit, Rpc19 in Pol I and Pol III or Rpb11 in Pol II, which is a functional equivalent to the α_2_ homodimer in prokaryotes. Additionally, five small subunits of the core, Rpb5, Rpb6, Rpb8, Rpb10 and Rpb12, are shared by all three polymerases. These small subunits have no known equivalent in the eubacterial enzyme. They are conserved in a single RNA polymerase from Archaea, but a homologue of Rpb8 has been identified only in some archaeal species (2,3). Common small subunits either bind or bridge catalytic subunits that divide the polymerase core into interacting subassemblies (4).

Pol III is the largest of the three polymerases. It has additional distinctive subunits at the periphery of the core of the enzyme. They form Pol III-specific subcomplexes, Rpc82–Rpc34–Rpc31 and Rpc53–Rpc37, that function in the initiation and termination of transcription (for review, see (5)).

A hypothetical model of Pol III assembly is based on the relatively well-recognized analogous process for prokaryotic RNA polymerase. It starts with formation of the αα dimer, which interacts with the β subunit, followed by β′ subunit recruitment (6). The existence of intermediate complexes in the process of Pol III assembly was suggested by the mass spectrometry analysis of Pol III disassembly (7,8). These analyses revealed a stable Rpc128–Rpc40–Rpc19–Rpb12–Rpb10 subcomplex (analogue of ααβ bacterial core subcomplex) and a stable Rpc160–Rpb8–Rpb5 subcomplex (β′-like module), suggesting their formation in the initial step of complex assembly. The relatively easy *in vitro* dissociation of Rpc82–Rpc34–Rpc31 and Rpc53–Rpc37 modules from Pol III suggests that peripheral subunits are added as Pol III-specific subcomplexes later during Pol III assembly.

Rpb10 is a small 70-amino-acid subunit that is conserved from Archaea to eukaryotes, including humans, which is required for assembly of yeast Pol III and Pol I (9,10). Rbp10 over-expression suppresses conditional *rpc40* and *rpc19* mutations that prevent enzyme assembly (9), as well as a conditional *rpc128*-*1007* mutant that is located in the Rpc128 subunit near contact points for the association between Rpc128 and the Rpc40–Rpc19 heterodimer (11).

Numerous studies of Pol II complex biogenesis (for review, see (4)) have led the development of a model in which Pol II is assembled in the cytoplasm with help from assembly factors and transported to the nucleus as a complex together with a specific adaptor. Following dissociation from Pol II in the nucleus, the adaptor is exported back to the cytoplasm. Pol III core enzymes probably utilize a similar assembly pathway. Several factors, such as Bud27, an unconventional prefoldin protein (12,13), the putative GTP-ases Gpn2 and Gpn3 (14), and the assembly/import factor Iwr1 (15), are common to Pol II and Pol III biogenesis.

A set of Pol III subunits show coordinated nuclear import indicating that the Pol III core is assembled in the cytoplasm, with additional components binding in the nucleus (16). This biphasic the assembly of Pol III likely requires specific auxiliary proteins. A candidate auxiliary protein is Rbs1, which was also identified in the genetic screen for suppressors of the Pol III assembly mutant *rpc128*-*1007*, in which Rpb10 was also selected (11). Genetic suppression correlated with higher levels of tRNA transcription, an increase in the stability of Pol III subunits, and their stronger interaction. Additionally, Rbs1 physically interacted with a subset of Pol III subunits (i.e., Rpc19, Rpc40 and Rpb5) and the Crm1 exportin. We postulated that Rbs1 binds to the Pol III complex or subcomplex and facilitates its translocation to the nucleus (11).

We postulated that the key role of Rbs1 might lie in increasing the expression of Rpb10. The results showed that Rbs1 indeed regulates the steady-state levels of *RPB10* mRNA, binding directly to the 3′ untranslated region (UTR). However, Rbs1 also binds to the 3′UTRs of other mRNAs *in vivo*. Moreover, Rbs1 forms a complex with the helicase Upf1, which is similarly involved in controlling *RPB10* mRNA levels. RNA binding requires the R3H domain present in the N-terminal part of Rbs1 protein. We identified Rbs1 homologs in other eukaryotes, including human R3H domain protein 2 (R3HDM2), which is known to interact with mRNA (17), suggesting that the regulatory mechanism we identified may also operate in higher organisms.

## MATERIALS AND METHODS

### Strains, media and growth conditions

**Figure 1.**
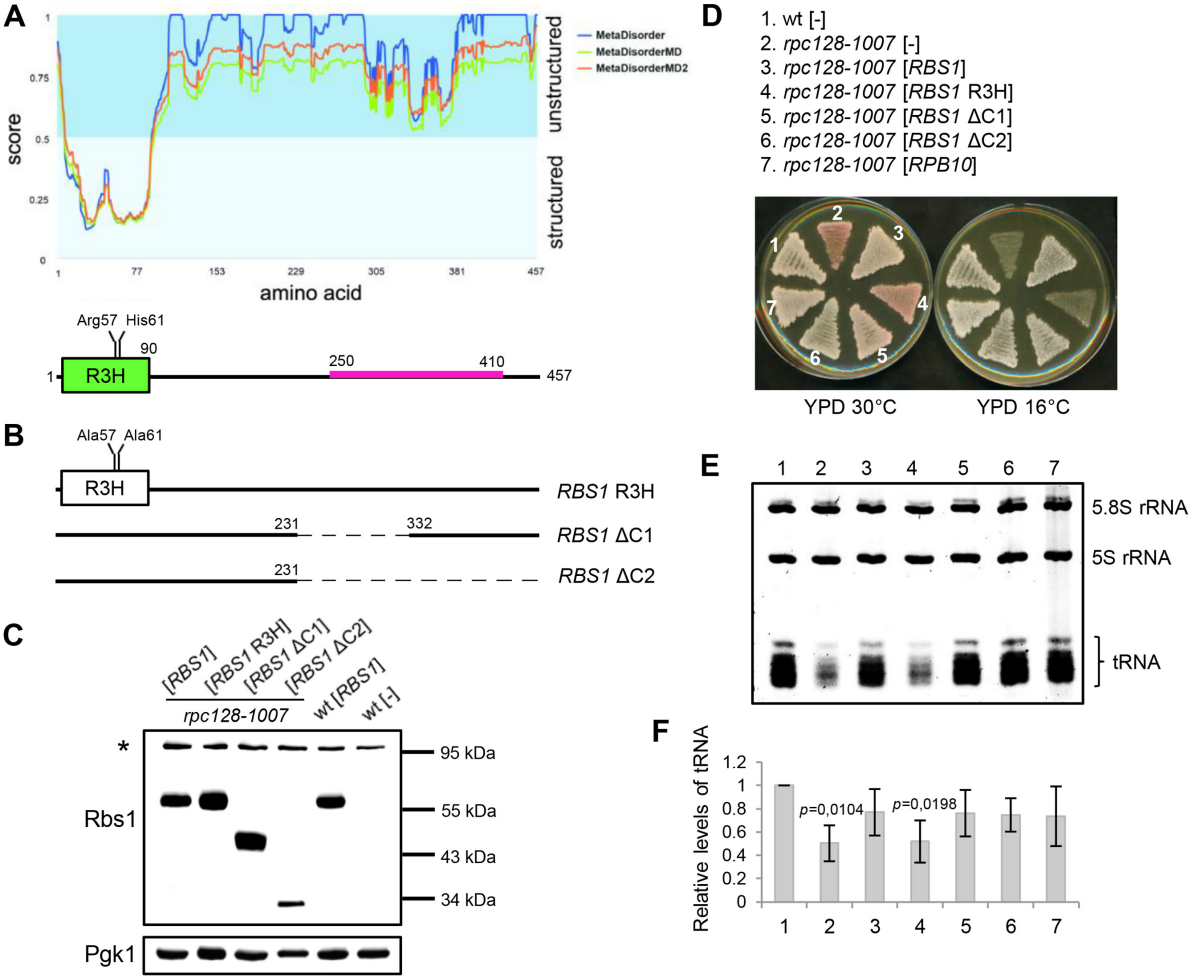
The R3H domain is essential for the function of Rbs1 protein in Pol III assembly. (**A**) Predicted disorder of Rbs1 protein according to Metadisorder server (30). Localization of the R3H domain (green) and the prionogenic sequence (pink) is shown below. (**B**) Schematic presentation of modified versions of *RBS1* that were constructed on corresponding plasmids: [*RBS1* R3H], [*RBS1* ΔC1] and [*RBS1* ΔC2]. See the Materials and Methods section for details. (**C-F**) Examination of transformants of control strain (wild type [wt]) and *rpc128*-*1007* mutant with plasmids that encoded modified versions of Rbs1 protein, [*RBS1*] and [*RPB10*] control plasmids, and the empty vector [–]. (**C**) The modified versions of Rbs1 protein were efficiently expressed. Yeast cells were analyzed by western blot. Antibody specific for Rbs1 detects only overproduced protein. Determination of Pgk1 levels served as loading control. (**D**) Inactivation of the R3H domain prevented genetic suppression of the Pol III assembly mutant. Cells that were grown on an SC-ura plate were replicated on YPD plates and incubated for 3 days at the respective temperatures. (**E**) Inactivation of the R3H domain prevented the correction of low tRNA levels in the Pol III assembly mutant. Small RNA species were separated on a 7 M urea–6% polyacrylamide gel using equal amounts of RNA per lane (5 μg) and stained with ethidium bromide. (**F**) Bands corresponding to total tRNAs were quantified. Bars represent tRNA levels normalized to 5.8S rRNA which served as loading control. Standard deviations were estimated on the basis of three independent experiments. The *P* value calculated for ratio of tRNAs (*rpc128*-*1007*[-]/wt[-], *rpc128*-*1007*[*RBS1*R3H]/wt[-] showed statistical significance (*P* < 0.02). *P* values were calculated using a two-tailed *t*-test.

**Figure 2.**
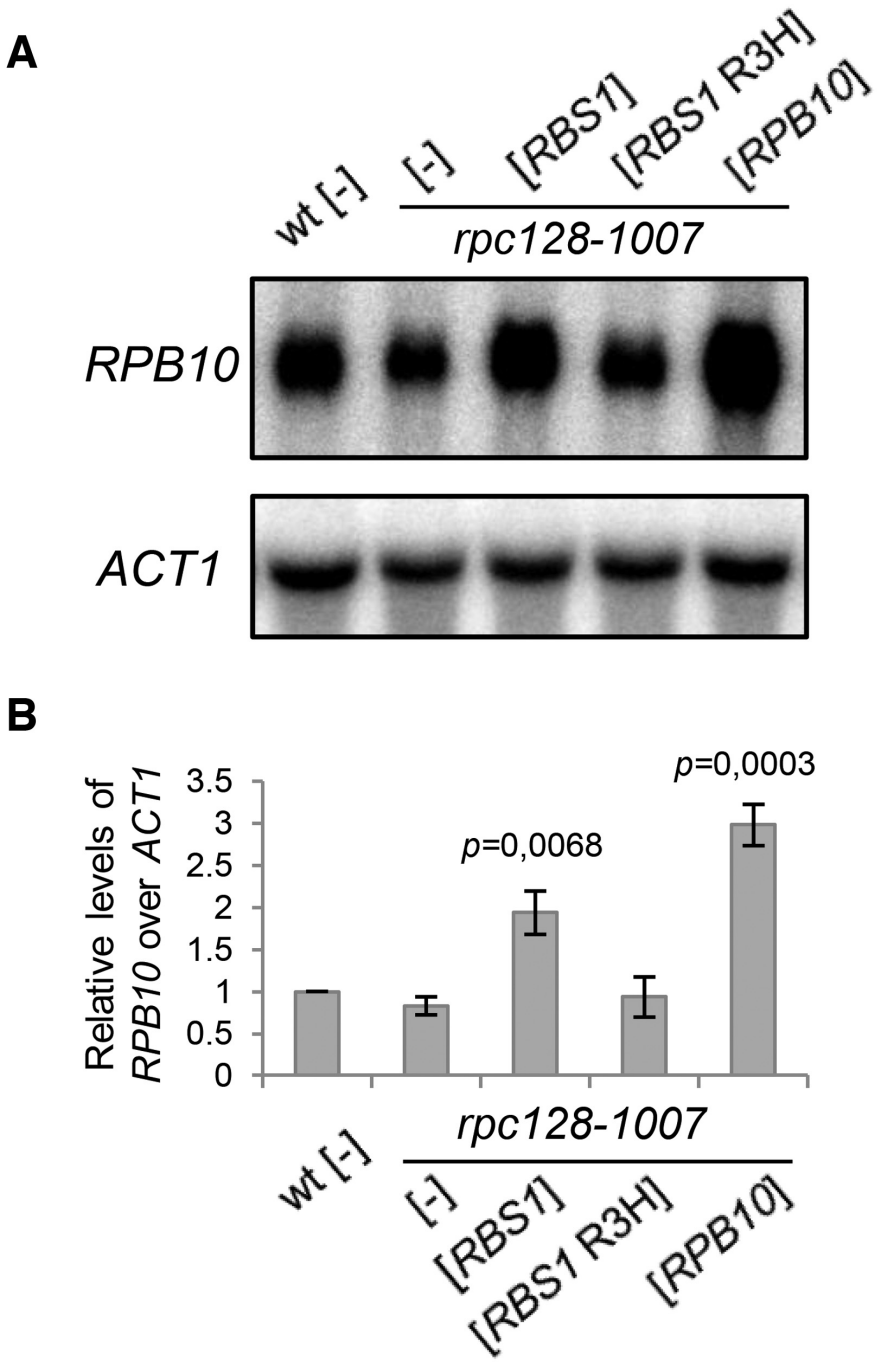
Effect of *RBS1* overexpression on steady-state levels of *RPB10* mRNA. (**A**) RNA that was isolated from the control strain (wt) and transformants of the *rpc128*-*1007* mutant with the [*RBS1* R3H] plasmid that encoded the mutated Rbs1 R3H protein, [*RBS1*] and [*RPB10*] control plasmids, and the empty vector [–] was analyzed by northern blot using probes that were specific to *RPB10* mRNA and *ACT1* mRNA that encodes actin (loading control). (**B**) The levels of *RPB10* mRNA were normalized to the loading control and calculated relative to levels in the wt strain, which was set to 1. Bars represent the mean ± standard deviation of three independent experiments. *P* values were calculated using a two-tailed *t*-test.

**Figure 3.**
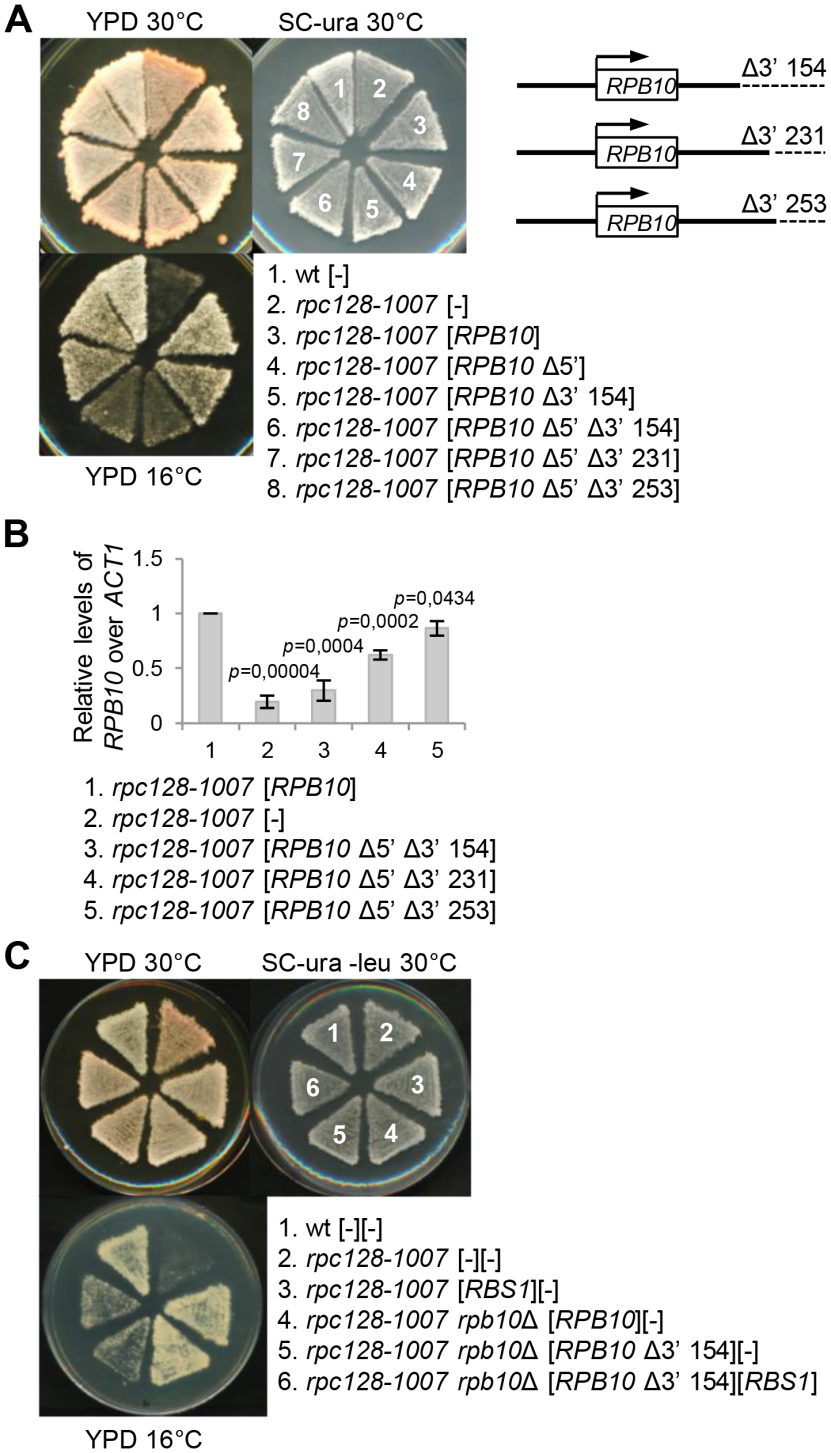
The role of Rbs1 in controlling Pol III assembly is supported by the 3′ regulatory region of the *RPB10* gene. The modified versions of the *RPB10* gene that lacks designated sequences in the 5′ and/or 3′ regulatory regions were constructed on the plasmids as described in Supplementary data. (**A**) Effect of deletions in the 3′ UTR on suppression of the *rpc128*-*1007* Pol III assembly mutant by *RPB10*. Δ3′ 154, Δ3′ 231 and Δ3′ 253, respectively, limited 3′UTR to 154, 231 and 253 nucleotides downstream a stop codon. The control strain (wt) and transformants of the *rpc128*-*1007* mutant with derivatives of [*RPB10*] containing designated deletions, the [*RPB10*] control plasmid, and the empty vector [–] were grown on SC-ura plates, replicated on YPD plates, and incubated for 3 days at the respective temperatures. (**B**) RNA isolated from transformants of the *rpc128*-*1007* mutant with derivatives of [*RPB10*] containing designated deletions and the [*RPB10*] control plasmid was used to synthesis of cDNA samples that were analyzed by RT-qPCR. mRNA levels were normalized to *ACT1* mRNA and calculated relative to amounts in the strain harboring the [*RPB10*] control plasmid, which was set to 1. Bars represent the mean ± standard deviation of three independent experiments. *P* values were calculated using a two-tailed *t*-test. (**C**) Δ3′154 deletion in 3′ UTR of *RPB10* negatively affected suppression of the Pol III assembly mutant *rpc128*-*1007* by *RBS1*. A double *rpb10*Δ *rpc128*-*1007* mutant that harbored the [*RPB10* Δ3′154] plasmid was additionally transformed with the [*RBS1*] plasmid or empty vector [–]. A double *rpb10*Δ *rpc128*-*1007* mutant that harbored [*RPB10*], a single *rpc128*-*1007* mutant that harbored [*RBS1*], and the wild type strain (wt) were additionally transformed with the respective empty vectors and served as controls. Yeast cells that were grown on an SC-ura-leu plate were replicated on YPD plates and incubated for 3 days at the respective temperatures.

The *rpc128*-*1007* mutant (MJ15-9C MATa *SUP11 ade2*-*1 ura3*-*1 lys2*-*1 leu2*-*3,112 his3*) and isogenic wild type strain MB159-4D, described previously (11), were used for experiments presented in Figures 1–3. *rpc128*-*1007* was also used to generate isogenic *upf1*Δ *rpc128*-*1007* mutant (Figure 6). *rbs1*Δ and *upf1*Δ are derivatives of wild type BY4741 strain (Euroscarf). The following media were used for growing yeast: YPD (2% glucose, 2% peptone, 1% yeast extract) and SC (2% glucose, 0.67% yeast nitrogen base without amino acids). SC-ura, SC-leu or SC-ura-leu contained 20 μg/ml of the amino acids required for growth, except for uracil, leucine or both, respectively. The start liquid cultures were grown overnight in SC-ura SC-leu or SC-ura-leu, transferred to YPD and grown to log phase (OD_600_ = 0.6). For experiments presented on Figures 1E–F, 2, 3B and 6A–C cells in log phase were transferred to 16°C for two hours. Details concerning strain construction and specific media were described in Supplementary data.

### Plasmids

YEp181-*RBS1* (2μ, *LEU2*) and pRS316-*RPB10* (*CEN6*, *URA3*) called here [*RBS1*] and [*RPB10*], respectively were described previously (11). The following derivatives of YEp181-*RBS1* were used: [*RBS1* R3H], constructed by substitution of conserved Arg57Ala and His61Ala; [*RBS1*-Myc] and [*RBS1* R3H-Myc], containing the wild type or mutated version of *RBS1* tagged with Myc epitope at the 3′ termini; [*RBS1* ΔC1] and [*RBS1* ΔC2], constructed by deletion of fragments of *RBS1* encoding 231–332 aa and 231–457 aa, respectively. Derivatives of [*RPB10*], [*RPB10* Δ5′], [*RPB10* Δ3′ 154], [*RPB10* Δ5′Δ3′ 154], [*RPB10* Δ5′Δ3′ 231], [*RPB10* Δ5′Δ3′ 253] contain partial deletions of 5′ and/or 3′ regulatory sequences of the *RPB10* gene. pRS316-HA-*RPB10* contains the HA-epitope sequence fused at N-terminus of *RPB10* gene with 5′ and 3′ regulatory regions. See Supplementary data for construction details.

### Isolation of protein-bound poly(A) mRNA

Cells were resuspended with 25 ml of phosphate buffered saline (PBS) containing 0,005% NP40 and transferred to a 10 cm Petri dish and placed on ice. Dishes were exposed to 254 nm UV in a UV crosslinker with 400 mJ/cm^2^ (18). Then cells were harvested by centrifugation at 4,000 rpm for 2 min at 4°C and resuspended in 0,5 ml of lysis buffer (100 mM Tris–HCl pH 7.5, 500 mM LiCl, 10 mM EDTA, 1% TritonX-100, 5 mM DTT, 20 units/ml DNase I [Thermo scientific], 100 units/ml RiboLock RNase inhibitor [Thermo Scientific], complete EDTA-free protease inhibitor cocktail [Roche]) (19). Cells were disrupted in the presence of glass beads at 4°C using a Vibramax disruptor (GENE) in 6 cycles (30 s of disruption, 30 s incubation on ice). After punched a hole in the bottom of the tube with needle the lysate was transferred to a fresh 1.5 ml tube by spin down and was passed five times through a needle to break the chromatin and clarified by a 10 min centrifugation at 14,000 rpm at 4°C. Protein concentration was measured by Bradford assay (Bio-Rad). 200 μl (1 mg) of Dynabeads oligo(dT)_25_ (Ambion) were washed according to the manufacturer's instructions and mixed with the 1.5 mg of lysate. The mixture was incubated at room temperature for 40 min with gentle stirring. Then oligo(dT)_25_ beads were washed two times with buffer A (10 mM Tris–HCl pH 7.5, 150 mM LiCl, 1 mM EDTA, 1% SDS) and two times with buffer B (10 mM Tris–HCl pH 7.5, 150 mM LiCl, 1 mM EDTA). Elution was performed by adding 30 μl of 10 mM Tris–HCl, pH 7.5 at 80°C for 2 min with shaking (1000 rpm) in a mixer. 15 μl of sample was separated by 10% SDS-PAGE and analyzed by western blotting.

### CRAC analysis

The CRAC (UV-crosslinking and analysis of cDNA) analysis was performed as previously described (20–22). In brief, following cell lysis, protein–RNA complexes were isolated by three-step purification (IgG column, TEV elution and Ni-NTA column). RNA was mildly digested with RNase A + T1 after first purification step, and linkers were ligated on Ni-NTA beads. Protein–RNA complexes were recovered by SDS-PAGE, reverse transcribed and PCR amplified for Illumina sequencing.

### Upf1-TAP and Rbs1-Myc co-immunoprecipitation

Cells were resuspended in lysis buffer (20 mM HEPES pH 7.4, 145 mM KCl, complete EDTA-free protease inhibitor cocktail [Roche]) and lysed by shaking with glass beads for 30 min at 4°C. The lysate was cleared by centrifugation (20 min at 4°C and 14 000 rpm). Protein concentration of extracts were determined with Bradford assay (Bio-Rad). Extracts were divided into two equal parts, one part was treated with RNase A and RNase T1 and the other part was not. Immunoprecipitations were done in parallel. Extract was treated first with 25 units of RNase A (Thermo scientific) and 500 units/ml of RNase T1 (Thermo scientific) and 20 min on ice, before immunoprecipitation. The sample was also treated a second time with RNase A and RNase T1, after adsorption of proteins on beads and washing (23). Dynabeads PanMouse IgG magnetic beads (Invitrogen) suspension were washed two times with 0.1% bovine serum albumin in PBS and incubated with 1 mg of protein extract for 1 h at 4°C with gentle shaking. Beads were collected with a magnet and washed two times with a first buffer A (20 mM HEPES pH 7.4, 145 mM KCl, 0.1% NP40), next incubated second time with 750 units/ml of RNase A and 3000 units/ml of RNase T1 in buffer B (20 mM HEPES pH 7.4, 145 mM KCl, 1 mM DTT) for 20 min at 25°C and washed again two times with buffer B. Elution of immunoprecipitated proteins from the beads was performed by adding 25 μl of 1% SDS at 65°C for 10 min with shaking (1000 rpm) in a mixer. 13 μl of sample was separated by 6% SDS-PAGE and analyzed by western blotting.

RNA isolation and northern hybridization; cDNA synthesis and reverse transcription-quantitative PCR (RT-qPCR); protein extraction and western blot analysis were described in the supplementary data. The primer sequences used for northern hybridization and RT-qPCR are listed in the Supplementary Tables S1 and S2.

### Search for homologs of Rbs1

In order to find homologs of Rbs1 across different kingdoms, PSI-BLAST searches were performed with sequences of Rbs1, maize DIP1 and human RH3 protein (R3H domain-containing protein 2, sequence ID: XP_011536342.1) as queries. Four rounds of PSI-BLAST was performed with the sequence number limit for each round set to 500. In the search with human protein sequence query, all 500 sequences hits were from vertebrates. They were essentially identical (similarity of 95% or higher), with the exception of a sequence from North Island brown kiwi (*Apteryx mantelli*, XP_013815153.1). For further analysis we used R3H domain-containing protein 2 from human and arbitrarily chosen sequences from *Xenopus leavis* (Xl) and *Danio rerio* (Dr). Next, we performed a PSI-BLAST search with Rbs1 as a query against the non-redundant database of nucleotide sequences from animals excluding vertebrates, which retrieved multiple sequences from invertebrates, including *Drosophila* encore. Searches with Rbs1 and DIP1 sequences as queries found 500 homologues form various fungal and plant species, respectively. The sequences from each search were aligned and their redundancy was reduced using Expasy Decrease Redundancy server with similarity threshold set to 60%. The regions corresponding to R3H-SUZ domains were extracted and sequences from all PSI-BLAST searches combined. The final alignment was performed using Promals3D (24) and sequence redundancy was reduced again with similarity threshold of 60%. Phylogenetic trees were generated using phylogeny.fr server (25,26).

## RESULTS

### The R3H domain supports the function of Rbs1 in controlling Pol III assembly

We explored specific features of Rbs1 protein and their contributions to Pol III assembly. The Rbs1 protein sequence is composed of two regions; the N-terminal portion has a highly ordered structure while the C-terminal region is mostly disordered (Figure 1A). The N-terminal portion comprises the well-defined R3H domain (residues 1–90; (27)) which has been structurally characterized. A crystal structure of the RNA-interacting R3H domain from human poly(A)-specific ribonuclease (PARN) was previously solved (28). A nuclear magnetic resonance (NMR) structure of the R3H domain from human Smubp-2 protein was also reported (29). The domain comprises an antiparallel three-stranded β-sheet with two α-helices on one side of the sheet. The helices are located at the N-terminus and between the first and second β-strands. Conserved arginine and histidine residues that are separated by three amino acids (Arg57 and His61 in Rbs1), from which the domain derives its name, are located in the second helix. Based on the results from MetaDisodered server (30), Rbs1 is predicted to be disordered after residue 90. However, the 170–250 region of residues encompasses several predicted secondary structural elements. After residue 250, very few secondary structures are predicted to form, and the Rbs1 sequence is expected to be highly disordered. Additionally, a proteome-wide study identified a prionogenic (aggregation-promoting) sequence in Rbs1 between residues 250 and 410 (31).

To determine the importance of the regions of Rbs1 protein that are mentioned above, we constructed several modified versions of *RBS1* (Figure 1B). We first generated the *RBS1* R3H mutant allele, in which the conserved R57 and H61 residues that are located within the R3H domain were changed to alanine. The mutant version of the protein, referred to as Rbs1 R3H, is predicted to adopt a native-like conformation but should have a substantially compromised ability to bind RNA. Next, we created two deletions of the 3′-terminal part of the *RBS1* open reading frame, designated *RBS1* ΔC1 and *RBS1* ΔC2, that encoded shorter versions of Rbs1 protein: Rbs1 ΔC1 of 356 aa (deletion of 231–332 aa fragment) and Rbs1 ΔC2 of 230 aa (deletion of 231–457 aa fragment). All of mutated *RBS1* alleles retained the original reading frame and the modified versions of Rbs1 protein were efficiently expressed (Figure 1C).

The effects of these mutations on the activity of Rbs1 were verified by complementation of the cold-sensitive *rpc128*-*1007* mutation in the second largest subunit of Pol III, which causes a defect in assembly of the polymerase complex (Figure 1D). The *rpc128*-*1007* strain carried additional *SUP11* and *ade1*-*2* mutations that allowed us to monitor the tRNA-dependent phenotype according to the colony color. This was possible because the presence of the *ade2*-*1* nonsense mutation led to pigment accumulation when the dosage of the suppressor tRNA *SUP11* (Tyr/UAA) was low. The low *SUP11* dosage and resulting red colony color were presumed to indicate that the global low tRNA levels were a consequence of the Pol III assembly defect in *rpc128*-*1007* cells (Figure 1D, strain 2). Complementation by the native *RBS1* and *RPB10* genes that were selected previously in the screen for dose suppressors of the *rpc128*-*1007* mutation (11) resulted in a white colony color, and growth at low temperature served as positive controls of suppression (Figure 1D, compare strain 2 and strains 3 and 7).

Clearly, the *RBS1* R3H mutant allele was unable to overcome both the colony-color and cold-sensitive phenotypes of the *rpc128*-*1007* mutant, thus demonstrating significance of the R3H domain of Rbs1 for genetic suppression (Figure 1D, compare strains 2 and 4). In contrast, both deletions in the C-terminal region, *RBS1* ΔC1 and *RBS1* ΔC2, recovered the colony color and restored the growth of *rpc128*-*1007* cells at low temperature (Figure 1D; compare strain 2 and strains 5 and 6). These findings indicated that the N-terminal portion of Rbs1 with an active R3H domain was sufficient for genetic suppression of the *rpc128*-*1007* phenotype. The C-terminal, largely disordered portion of Rbs1 protein, including the prionogenic sequence, was not required for this suppression.

To investigate functional suppression of the *rpc128*-*1007* mutation, we examined whether the alleles of *RBS1* were able to correct the global low tRNA levels in *rpc128*-*1007* cells. The analysis of total RNA on ethidium bromide-stained gels (Figure 1E) and quantification of tRNA bands (Figure 1F) confirmed that the low tRNA levels in the *rpc128*-*1007* mutant were restored by the *RBS1* ΔC1 and *RBS1* ΔC2 alleles but not by the *RBS1* R3H allele (Figure 1F; compare lane 2 and lanes 4, 5, and 6). Although 5S rRNA is also a product of Pol III, its level is not affected in *rpc128*-*1007* cells or the other strains tested here. Many other Pol III mutants lead to a decrease of tRNA synthesis but do not alter the transcription of 5S rRNA (11,32). In summary, the N-terminal 1–230 aa fragment of Rbs1 that contains the R3H domain was essential for suppressing both genetic and molecular phenotypes of the *rpc128*-*1007* mutation and the function of Rbs1 protein in Pol III assembly.

### Interplay between Rbs1 and Rpb10 in controlling Pol III assembly

The functional RNA-interacting R3H domain of Rbs1 was essential for suppressing *rpc128*-*1007*, suggesting that Rbs1 may play an indirect regulatory role by binding RNAs. Since *RPB10* and *RBS1* were identified in the same over-expression screen for Pol III assembly defects (11), we tested whether Rbs1 affects *RPB10* mRNA levels.

The effect of Rbs1 overexpression on *RPB10* mRNA levels were examined by northern blot (Figure 2A). The overproduction of Rbs1 resulted in a stronger hybridization signal with the *RPB10*-specific probe. The steady-state level of *RPB10* mRNA was two-fold higher in the *rpc128*-*1007* mutant that carried a plasmid with the *RBS1* allele compared with the same mutant that was transformed with the control vector (Figure 2B). No effect of the *RBS1* R3H allele on *RPB10* mRNA levels in *rpc128*-*1007* cells was observed. These findings indicate that Rbs1 is involved in controlling steady-state levels of *RPB10* mRNA, and the R3H domain plays an important role in this regulation.

*RPB10* mRNA levels correlated with genetic and functional suppression of the Pol III assembly defect in *rpc128*-*1007* cells (compare Figures 1D, E and 2). Steady-state levels of *RPB10* mRNA are under the control of 5′ and 3′ regulatory sequences (UTRs); their length is estimated for 143 and 365 nucleotides, respectively (33).

The modified versions of the plasmid encoding *RPB10* with deleted fragments at 5′ and 3′ ends of the inserted gene were constructed to examine the role of the regulatory sequences in suppressing the *rpc128*-*1007* mutant (see Supplementary data for construction details). The efficiency of *rpc128*-*1007* suppression by *RPB10* was decreased by a deletion mutation designated Δ3′ 154 which limited the 3′UTR to 154 nucleotides downstream from the stop codon (Figure 3A, strains 5, 6). However, suppression was not disturbed by *RPB10* with longer 3′UTR, Δ3′ 231 and Δ3′ 253 (Figure 3A, strains 7, 8). A role for the 3′ UTR in controlling steady-state *RPB10* mRNA levels was confirmed by RT-qPCR. The amount of *RPB10* mRNA with the 3′UTR shortened to 154 nucleotides was decreased by 3.37±0.09-fold over the control (Figure 3B, compare lane 3 and 1) suggesting that the Δ3′ 154 deletion makes this transcript relatively unstable. Possibly it is not efficiently polyadenylated, since deleted fragment of 3′UTR included two potential polyadenylation signal sequences UAUAUA, localized 187 and 334 downstream of a stop codons. *RPB10* mRNA containing longer 3′UTRs, 231 or 253 nucleotides, were more abundant. *RPB10* mRNA levels in deletion mutants correlate with the efficiency of *rpc128*-*1007* suppression by *RPB10* (compare Figure 3A and B).

Next, we investigated the role of the 3′ UTR in *RPB10* in suppressing the *rpc128*-*1007* mutant by *RBS1* overdose. *RPB10* is an essential gene, but *rpb10*Δ that expresses *RPB10* with shortened 3′UTR from the [*RPB10* Δ3′154] plasmid is viable. Despite deletion of the chromosomal gene, weak suppression of the cold-sensitive *rpc128*-*1007* phenotype by [*RPB10* Δ3′154] was observed (Figure 3C, strain 5). Growth, however, was not improved by overexpression of the *RBS1* gene (Figure 3C, compare strains 5 and 6). These findings indicate that the 3′ UTR in the *RPB10* gene is required for efficient suppression of the *rpc128*-*1007* mutant by Rbs1.

Altogether, our results suggest that the participation of Rbs1 in Pol III assembly involves the control of steady-state levels of *RPB10* mRNA via its 3′ regulatory region.

### Rbs1 protein binds poly(A) mRNAs in an R3H domain-dependent manner

**Figure 4.**
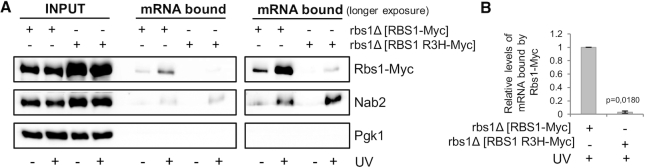
The binding of Rbs1 with poly(A) mRNA in living cells requires the R3H domain. (**A**) Poly(A) mRNA was isolated from cells that expressed Myc-tagged Rbs1 or Rbs1 R3H without or after RNA-protein cross-linking by UV irradiation. Input and poly(A) mRNA-bound fractions were analyzed by western blot with antibodies that were specific to Myc, Nab2 (positive control) and Pgk1 (loading control). mRNA bound proteins were visualized by shorter and longer gel exposure. Band intensities from western blot images were quantified using MultiGauge 3.0 software (Fujifilm). (**B**) The relative amount of mRNA bound in Rbs1-Myc was set to 1. Bars represent the mean ± standard deviation of three independent experiments. *P* values were calculated using a two-tailed *t*-test.

The importance of the R3H domain in Rbs1 suggests that the mechanism of action of Rbs1 may involve mRNA binding. Several proteins that contain the R3H domain have been previously shown to bind mRNA and regulate mRNA expression (34–36). We analyzed the association between Rbs1 and mRNAs in living cells after RNA-protein cross-linking by UV irradiation. *rbs1*Δ mutant cells that expressed Myc-tagged native Rbs1 or mutated Rbs1 R3H protein from the plasmid were UV irradiated, and poly(A) mRNA–protein complexes were affinity-purified using oligo(dT)_25_ beads. Proteins that were associated with mRNA were detected by western blot using Myc-specific antibody. Rbs1 bound with polyadenylated mRNA, whereas only a weak signal of mRNA-bound mutated Rbs1 R3H protein was observed, despite the fact that mutated protein was abundant in the whole cell extract (Figure 4). Rbs1 pull down was dependent on UV irradiation, confirming poly(A) mRNA binding by Rbs1. The known mRNA binding protein Nab2 (37) was used as a positive control. As expected, Nab2 recovery with poly(A) mRNA was dependent on UV irradiation but independent of the R3H mutation in Rbs1. Additionally, the samples were analyzed for the presence of Pgk1 protein, which is not expected to bind mRNA, to demonstrate that no contaminants were associated with the resin. Altogether, these data show that Rbs1 binds poly(A) mRNAs via the R3H domain.

### Rbs1 protein interacts with Upf1 helicase

**Figure 5.**
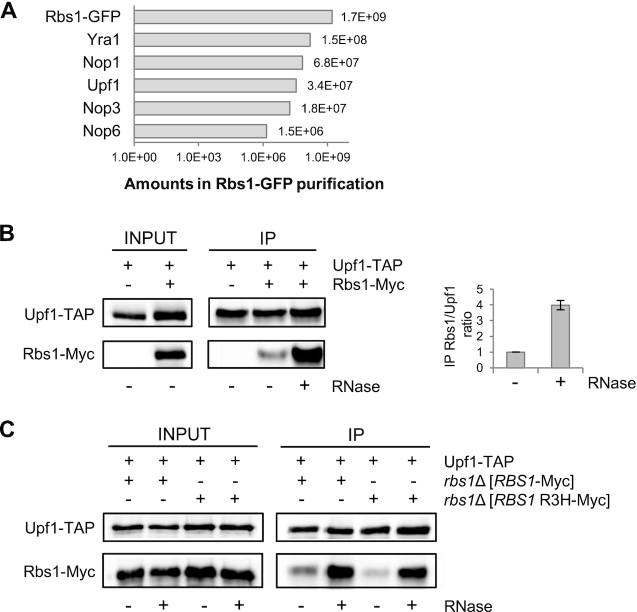
Rbs1 interacts with Upf1 helicase. (**A**) Upf1 is an RNA-interacting protein that was co-purified with Rbs1. The affinity purification of green fluorescent protein-tagged Rbs1 was followed by quantitative mass spectrometry. The relative amounts of co-purified proteins were determined by MaxQuant software, expressed as arbitrary units (11). (**B, C**) Upf1 and Rbs1 interaction, determined by co-immunoprecipitation. RNase treatment enhanced the Rbs1-Upf1 interaction. Total cell extracts (INPUT) were isolated from (**B**) a strain that expressed endogenous Myc-tagged Rbs1 and TAP-tagged Upf1 and from a control strain that expressed only Upf1-TAP or (**C**) the *rbs1*Δ strain that expressed TAP-tagged Upf1 and overexpressed Myc-tagged Rbs1 or Rbs1 R3H, encoded by multicopy plasmids. Extracts were subjected to immunoprecipitation using IgG-coated magnetic beads. This protocol was based on affinity of the protein A-containing TAP tag to IgG. Immunoprecipitated proteins were eluted and analyzed by western blot using peroxidase anti-peroxidase (PAP) and anti-Myc antibodies. RNase treatment of the extracts and beads is designated at the bottom of the western blot images. Band intensities were quantified using MultiGauge 3.0 software (Fujifilm). The ratio of immunoprecipitated Rbs1-Myc to Upf1-TAP from probes that were or were not treated with RNase was calculated. The ratio in the probe that was not treated with RNase was set to 1. Bars represent the mean ± standard deviation of two independent experiments.

Binding to polyadenylated RNA suggests the participation of Rbs1 protein in mRNA metabolism. Several RNA-interacting proteins were detected previously by affinity purification of tagged Rbs1, followed by quantitative mass spectrometry (11). Relatively high amounts of Rbs1-copurified Yra1, Nop1, Upf1, Nop3 and Nop6 RNA-interacting proteins were detected using MaxQuant software ((38); Figure 5A). Additionally, affinity purification coupled with mass spectrometry identified Rbs1 as an Upf1-interacting protein that is included in one of the two complexes that are involved in NMD (23). Thus, we further explored the interaction between Rbs1 and Upf1.

To confirm the interaction between Rbs1 and Upf1 *in vivo*, we used a strain that encoded functional Rbs1-Myc (11) and Upf1-TAP (23) tagged proteins that were expressed from the chromosomal loci. Both Upf1 and Rbs1 are RNA-binding proteins, and we investigated whether their interaction is RNA-dependent. The crude cell extract was divided into two equal parts. One part was treated with RNases, and the other part was not. Upf1-TAP was immunopurified from both parts of the extract with IgG-coated magnetic beads, and RNase treatment was repeated for the first part. Next, both immunoprecipitates were examined for the presence of Rbs1-Myc protein by immunoblotting. As shown in Figure 5B, Rbs1 selectively co-immunoprecipitated with TAP-tagged Upf1. Surprisingly, RNase treatment resulted in a stronger Upf1 interaction with Rbs1.

To further characterize the Rbs1-Upf1 association, we evaluated the role of the R3H domain in the Rbs1 protein. *rbs1*Δ mutant cells that expressed Upf1-TAP fusion and Myc-tagged native Rbs1 or mutated Rbs1 R3H protein from the plasmid were examined by immunoprecipitation as described above. Both wild type Rbs1 protein and mutated Rbs1 R3H protein interacted with Upf1 with the same efficiency. This interaction was stronger upon RNase treatment of the Upf1-TAP immunoprecipitates (Figure 5C). These findings indicate that Rbs1 and Upf1 proteins form the complex with each other irrespective to the functional R3H domain in Rbs1.

### Upf1 is involved in regulating *RPB10* mRNA levels and Pol III assembly

**Figure 6.**
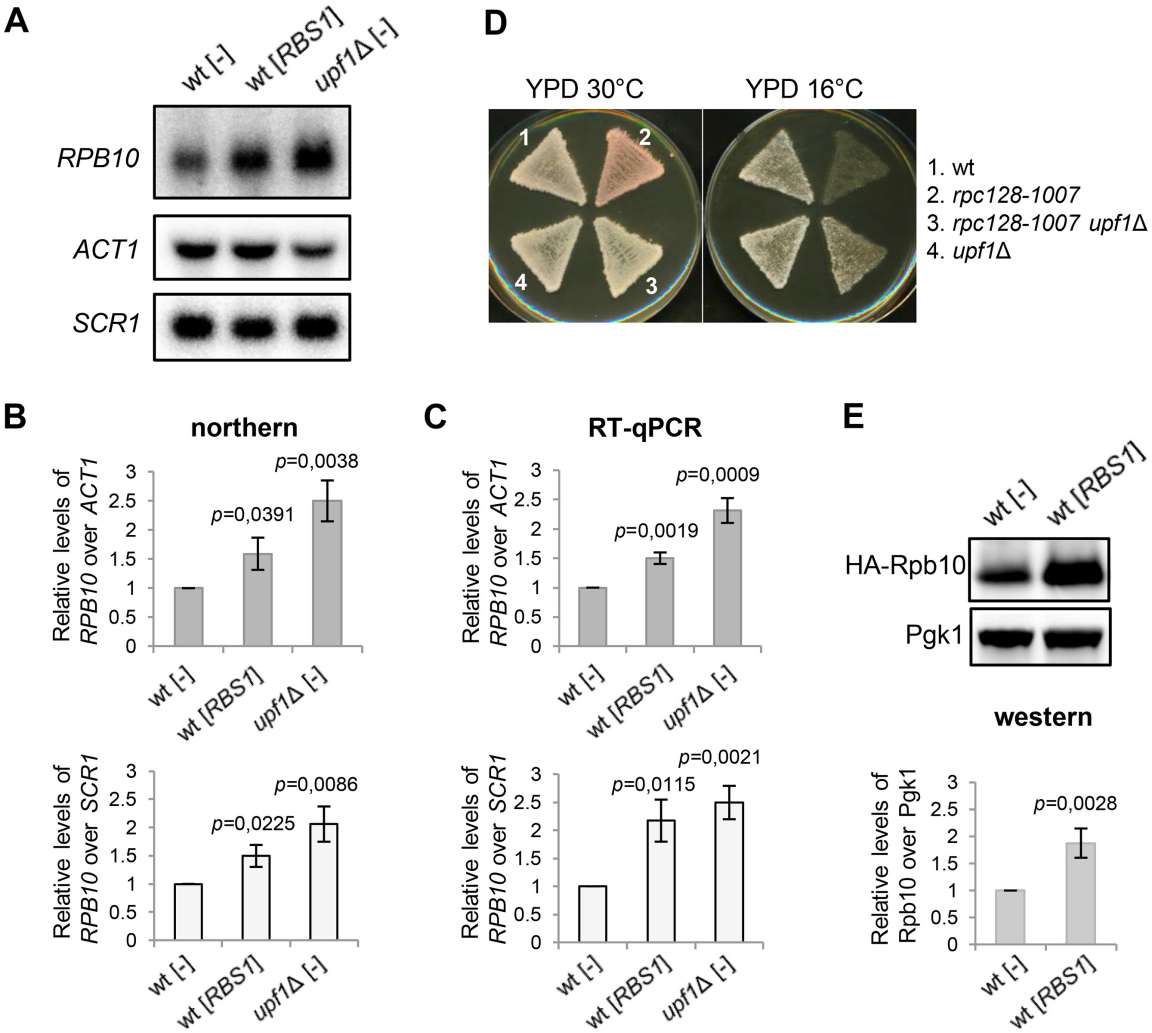
Effects of Upf1 and Rbs1 on *RPB10* expression and Pol III assembly. (**A–C**) Opposite effects of Rbs1 and Upf1 on *RPB10* mRNA levels. RNA was isolated from the *upf1*Δ mutant and a control strain (wt) that carried an empty vector [–] or the [*RBS1*] plasmid. RNA was independently analyzed by northern blot (**A, B**) and RT-qPCR (**C**). *RPB10* mRNA levels were normalized to *ACT1* mRNA (upper panel) or *SCR1* (lower panel *RPB10* mRNA levels were calculated relative to amounts in the wt strain, which was set to 1. (**D**) The phenotype of the Pol III assembly mutant *rpc128*-*1007* was suppressed by *upf1*Δ. Cells that were grown on YPD plates were replicated on fresh YPD plates and incubated for 3 days at the respective temperatures. (**E**) Protein extracts were prepared from a control strain (wt) that carried an empty vector [–] or the [*RBS1*] plasmid additionally transformed with a centromeric [HA-*RPB10*] plasmid. The band corresponding to HA-tagged Rpb10 was quantified, normalized to Pgk1 signal used as a loading control and calculated relative to amounts in the wt strain, which was set to 1. Bars represent the mean ± standard deviation of three independent experiments. *P* values were calculated using a two-tailed *t*-test (**B, C** and **E**).

Upf1 helicase is a principal regulator of NMD, a highly conserved mechanism for recognizing and rapidly degrading mRNAs that encode potentially harmful truncated proteins. In yeast, direct NMD targets typically have a premature termination codon or unspliced intron (39). Nonsense-mediated decay has also been reported to play a vital role in regulating wild type gene expression. It was previously shown to destabilize eukaryotic transcripts with upstream open reading frames or long 3′ UTRs (40–42). One common feature of mRNAs that are natural NMD substrates in yeast is a short coding region (41,43). Since *RPB10* mRNA encodes small 70-amino-acid protein, it is a potential candidate. Moreover, Upf1 formed a complex with Rbs1 protein (Figure 5), which controlled steady-state levels of *RPB10* mRNA in the *rpc128*-*1007* mutant (Figure 2). Hence, NMD may play a role in regulating *RPB10* expression. Supporting this possibility, the examination of wild type cells revealed opposite effects of Upf1 and Rbs1 on the accumulation of *RPB10* mRNA (Figure 6A and B). The level of *RPB10* mRNA was over two-fold higher in the *upf1*Δ mutant, suggesting that Upf1 may be involved in degrading this mRNA. The effect of *upf1*Δ on steady-state levels of *RPB10* mRNA was additionally confirmed by RT-qPCR (Figure 6C). *RPB10* mRNA levels were normalized to *ACT1* mRNA (upper panel) or *SCR1* (lower panel) commonly used as a loading controls in studying NMD-dependent mRNA decay (41,43). Next, we evaluated whether the increase in *RPB10* mRNA correlates with suppression of the Pol III assembly mutant *rpc128*-*1007* (Figures 1D and 2). As expected, *upf1*Δ deletion restored the growth of *rpc128*-*1007* cells at low temperature (Figure 6D, compare strains 2 and 3). These data indicate the regulatory role of Upf1 in Pol III assembly and support the physiological importance of the Upf1 interaction with Rbs1.

Higher levels of *RPB10* mRNA after *RBS1* overexpression suggest that *RPB10* mRNA is protected from degradation and more efficiently translated. To estimate levels of Rpb10 protein, cells overproducing Rbs1 were transformed by the plasmid containing HA- epitope sequence fused at 5′ terminus of *RPB10* gene with a long 3′ regulatory region and examined by western blotting with HA-specific antibody (Figure 6E). Quantification of the band corresponding to HA-tagged Rpb10 revealed 1.88±0.27-fold increase upon Rbs1 overexpression. We thus concluded that up-regulation of *RPB10* mRNA level by Rbs1 leads to elevated level of Rpb10 protein as this is proposed as the active component that drives Pol III assembly (11).

The level of Rpb10 protein in the *upf1*Δ mutant was also increased, but the abundance varied significantly between biologically independent experiments. Moreover, an additional band was observed on western blots suggesting Upf1-dependent regulation of *RPB10* expression translational or posttranslational level. Interpretation of these effects is not straightforward and needs further study.

**Figure 7.**
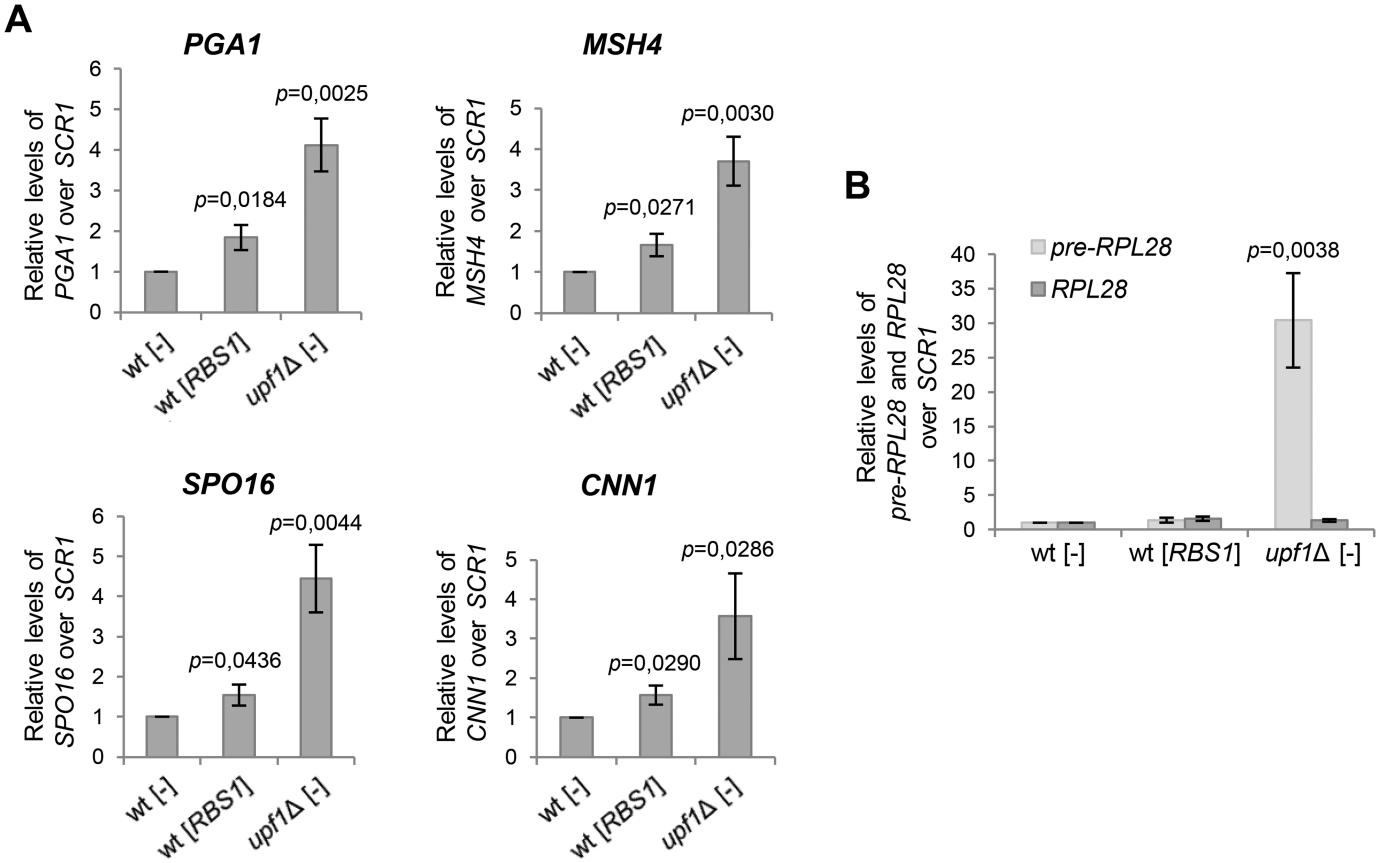
The RT-qPCR analysis indicated effects of Rbs1 on the expression of yeast genes that are controlled by 3′-UTR NMD decay (**A**) but no influence of Rbs1 on the level of *RPL28* pre-mRNA, a direct NMD target (**B**). RNAs that were isolated from the *upf1*Δ mutant and control strain (wt) that carried an empty vector [–] or [*RBS1*] plasmid were analyzed by RT-qPCR with probes that were specific to *PGA1* mRNA, *MSH4* mRNA, *SPO16* mRNA, and *CNN1* mRNA (A) or intron-containing *RPL28* pre-mRNA and mature *RPL28* mRNA (B). The levels of the tested mRNAs were normalized to *SCR1* RNA and calculated relative to levels in the wt strain, which was set to 1. Bars represent the mean ± standard deviation of three independent experiments. *P* values were calculated using a two-tailed *t*-test.

We explored the possibility that Rbs1, in addition to *RPB10* mRNA, is involved in controlling other Upf1-dependent transcripts. Based on previously published data (41,44) we selected transcripts of four genes for testing: *PGA1*, *MSH4, SPO16* and *CNN1*. The candidate genes were previously shown to be sensitive to NMD during vegetative growth, and changes in mRNA accumulation were functionally linked to their 3′ UTRs. We performed RT-qPCR to confirm expression levels of all of the tested transcripts in the *upf1*Δ deletion mutant. The fine tuning of *PGA1*, *MSH4, SPO16*, and *CNN1* expression by Rbs1 overproduction was observed (Figure 7). We also examined *RPL28* transcripts. The intron-containing precursor of *RPL28* mRNA is a known direct target of NMD, whereas mature *RPL28* mRNA is not (39). In contrast to the *upf1*Δ deletion, no effect of Rbs1 overproduction on *RPL28* pre-mRNA was observed (Figure 7B). The protection that is conferred by Rbs1 appears to involve only some NMD targets, possibly targets that are functionally linked to their 3′ UTRs.

### Rbs1 directly binds the 3′ UTRs of mRNAs

**Figure 8.**
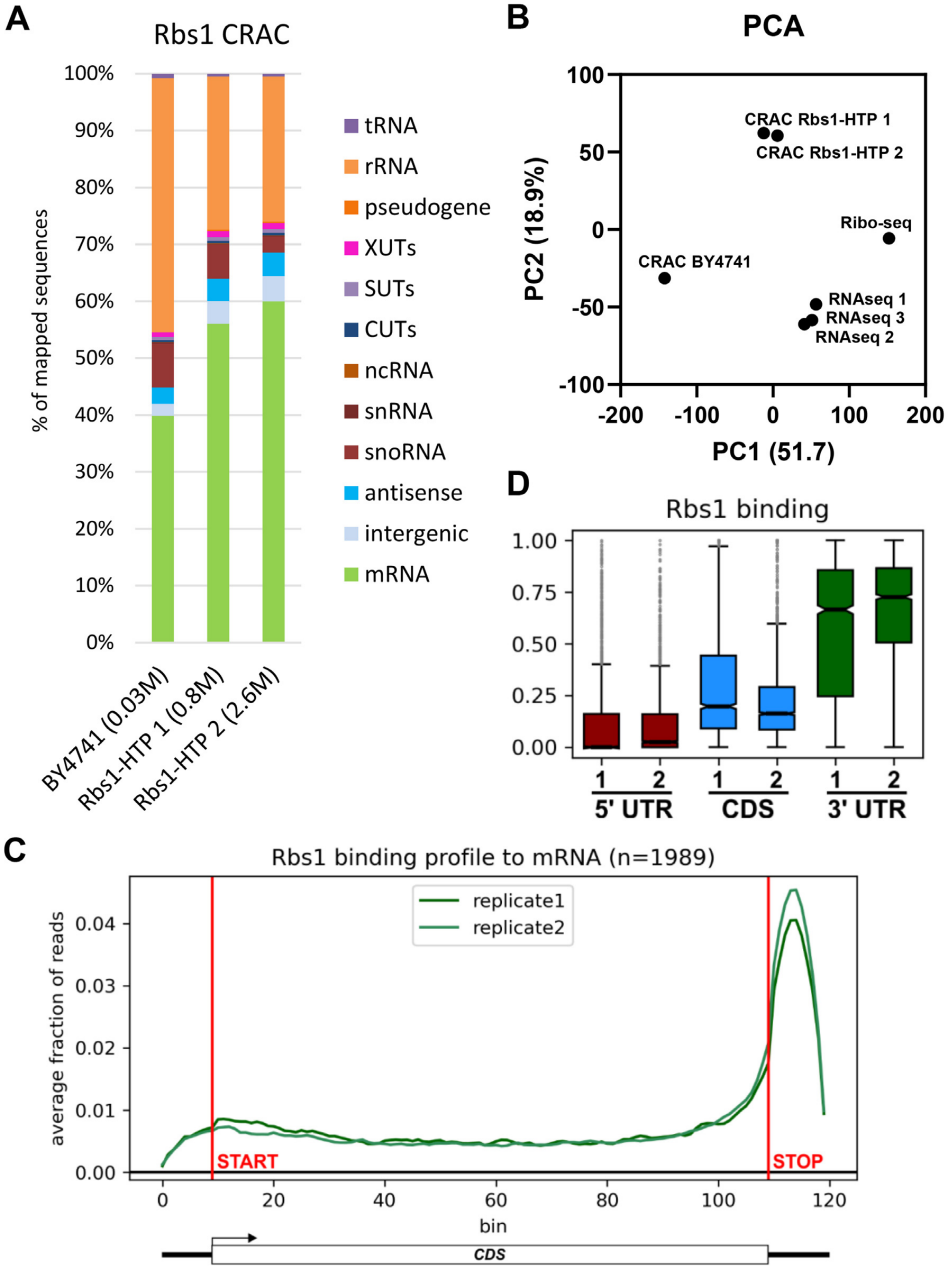
Rbs1 preferentially binds 3′UTR regions in mRNAs. (**A**) Transcriptome-wide binding profiles for Rbs1-HTP and for the control BY4741 strain expressing untagged Rbs1. Bar diagrams illustrate the percentage of all sequences mapped to each of the RNA classes indicated on the right of the figure. (**B**) Principal component analysis (PCA) showing differences between Rbs1 CRAC, RNA-seq and Ribo-seq. Axis titles show the extent of variation explained by a given principal component. (**C**) Metagene representation of read density over mature mRNAs (n = 1989). Data were separated into 120 bins: 10 for the 5′ UTR, 100 for the CDS and 10 for the 3′ UTR. Horizontal lines indicate were CDS starts and stops. Metagene analysis performed for mRNA containing at least 100 reads in Rbs1 CRAC data (*n* = 1989). (**D**) Boxplot of two Rbs1 CRAC replicates presenting binding to mature mRNA. Centre lines of box plots show the medians; box limits indicate the 25th and 75th percentiles; whiskers extend 1.5 times the interquartile range from the 25th and 75th percentiles, data points are plotted for outliers.

To confirm binding sites of Rbs1 in *RPB10* mRNA and identify genome-wide targets we employed UV-crosslinking and analysis of cDNA (CRAC). To allow CRAC Rbs1 was expressed as a fusion with a tripartite tag (His6–TEV protease cleavage site–protein A [HTP]) from the chromosomal locus, which was the only source of Rbs1 in the cell. Wild type, BY4741 strain, expressing untagged Rbs1 served as a negative control. Strains expressing Rbs1-HTP showed wild type growth rates, demonstrating that the fusion protein is functional. The CRAC analysis was performed as previously described (20–22). The sequencing reads were mapped to the genome to the define fraction of reads mapping to different classes of RNAs (Figure 8A). Analysis of the sequence data identified the major RNA target classes for Rbs1 binding as mature mRNA and rRNA, with lower association to other classes. Subsequent analyses were performed on reads mapped to the transcriptome. Two biological replicates presented good reproducibility (Supplementary Figure S1A) as shown by Spearman (*R*^Spearman^ = 0.82) and Pearson (*R*^Pearson^ = 0.97) correlation co-efficients.

To test the correlation level of Rbs1 CRAC data with mRNA abundance and translation level we compared the Rbs1 CRAC data with RNA-seq data (45) and ribosome profiling (Ribo-seq) data (46). In the PCA analysis, Rbs1 CRAC replicates cluster separately (Figure 8B), suggesting enrichment of specific transcripts. However good correlations, both with RNA-seq (*R*^Spearman^ = 0.7) and Ribo-seq (*R*^Spearman^ = 0.74) data (Supplementary Figure S1B) reveal dominant transcriptome-wide binding.

To define binding profile of Rbs1 we selected mRNAs with >100 reads per million (RPM) in CRAC data and constructed metagene profile (*n* = 1989). To normalize gene length, we divided each transcript into 120 bins: 10 for the 5′ UTR, 100 for CDS and 10 for the 3′ UTR. All reads mapping to a given transcript were normalized to 1, to construct an unbiased binding profile across all the analyzed transcripts. This analysis revealed that Rbs1 binding has a strong 3′ bias (Figure 8C), that remained clear when we analysed all yeast mRNAs (*n* = 6692) (Supplementary Figure S1 C). To confirm that the 3′ bias is directly due to binding within the 3′ UTRs, we calculated fraction of reads binding the 5′ UTR, CDS and the 3′ UTR for each transcript with more than 100 RPM (*n* = 1989). The analysis showed that nearly 75% of Rbs1 binding occurs within the 3′ UTR on mRNAs (Figure 8D).

**Figure 9.**
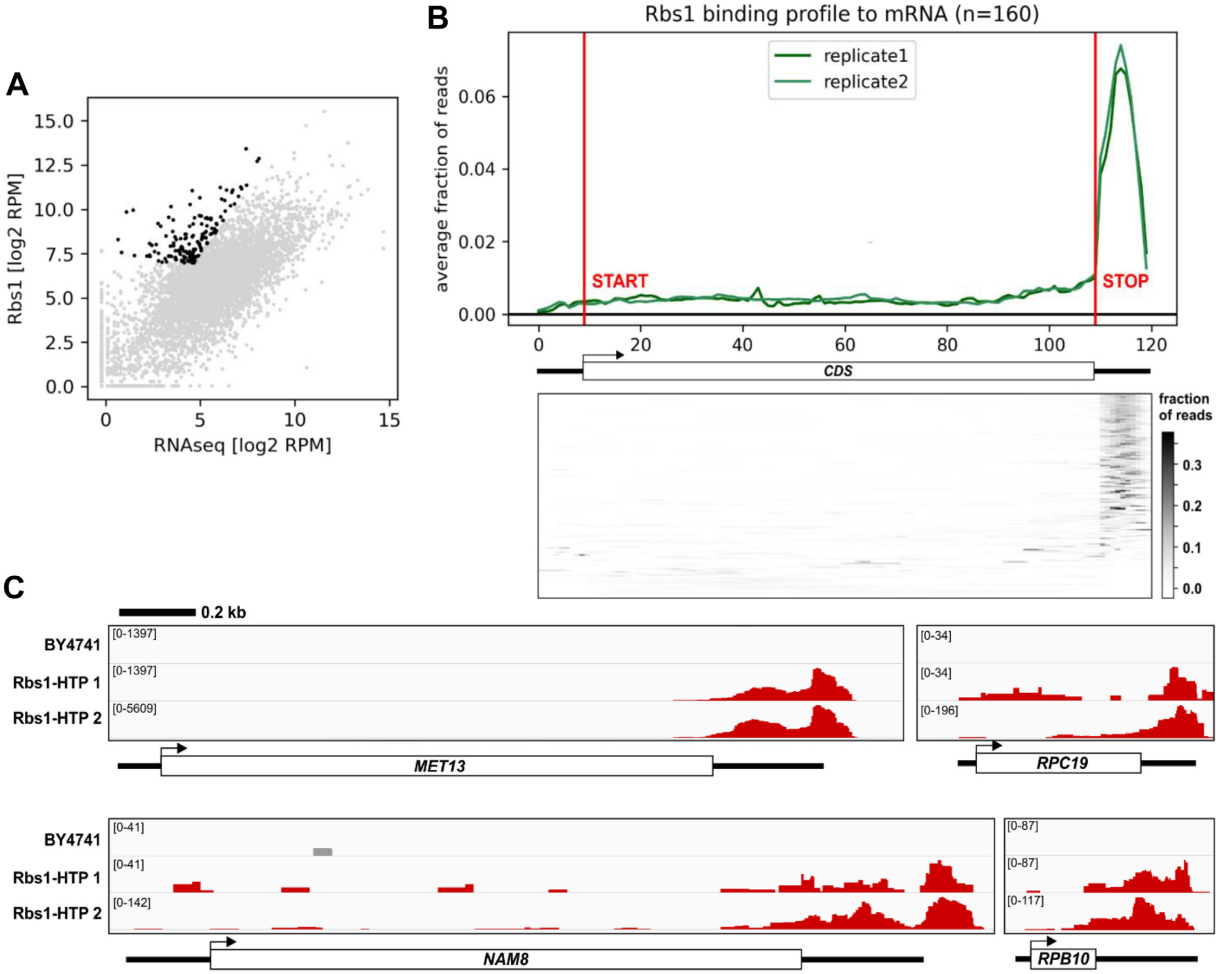
mRNAs enriched in Rbs1 binding. (**A**) Scatter plot comparing the Rbs1 RNA binding to RNA-seq with marked high confidence targets of Rbs1 (black). The targets were selected using following criteria: *P* value <0.01, ratio between Rbs1 binding and RNA-seq >1.5 and >128 uniquely mapped RPM. (**B**) Upper panel: Metagene representation of read density over high confidence Rbs1 target mature mRNAs (*n* = 160). Data were separated as panel 9C. Bottom panel: Heatmap representation of read density over high confidence Rbs1 target mature mRNAs (*n* = 160). (**C**) Binding of Rbs1-HTP across the *MET13, RPC19, NAM8* and *RPB10* mRNAs. Each track presents raw number of uniquely mapped reads, with the exact value indicated in the left part of each track. A scale bar is shown at the top.

Finally, we focused on mRNAs enriched in Rbs1 binding over RNAseq data. To do this, we applied three filtering criteria: (i) more than 128 RPM in Rbs1 CRAC data, (ii) 1.5-fold enrichment in Rbs1 CRAC data over RNA-seq and *P* value <0.01. To increase number of samples, and therefore statistical robustness of our analysis, we used two technical replicates of Rbs1 CRAC for each biological replicate. Application of filtering criteria allowed us to define high confidence targets of Rbs1 (*n* = 160) (Figure 9A). Rbs1 predominately binds targets in the 3′ UTR regions (Figure 9B) as showed by metagene profile and heatmap presenting profiles for individual transcripts. Notably, the list of high confidence Rbs1 targets include both *RPB10* (*P* value = 0.00159) and the mRNA encoding another Pol III subunit *RPC19* (*P* value = 0.000189).

Examples of single-gene tracks of Rbs1 binding to *MET13, RPC19, NAM8* and *RPB10* mRNAs are presented (Figure 9C).

### Evolutionary conservation of Rbs1 domains

**Figure 10.**
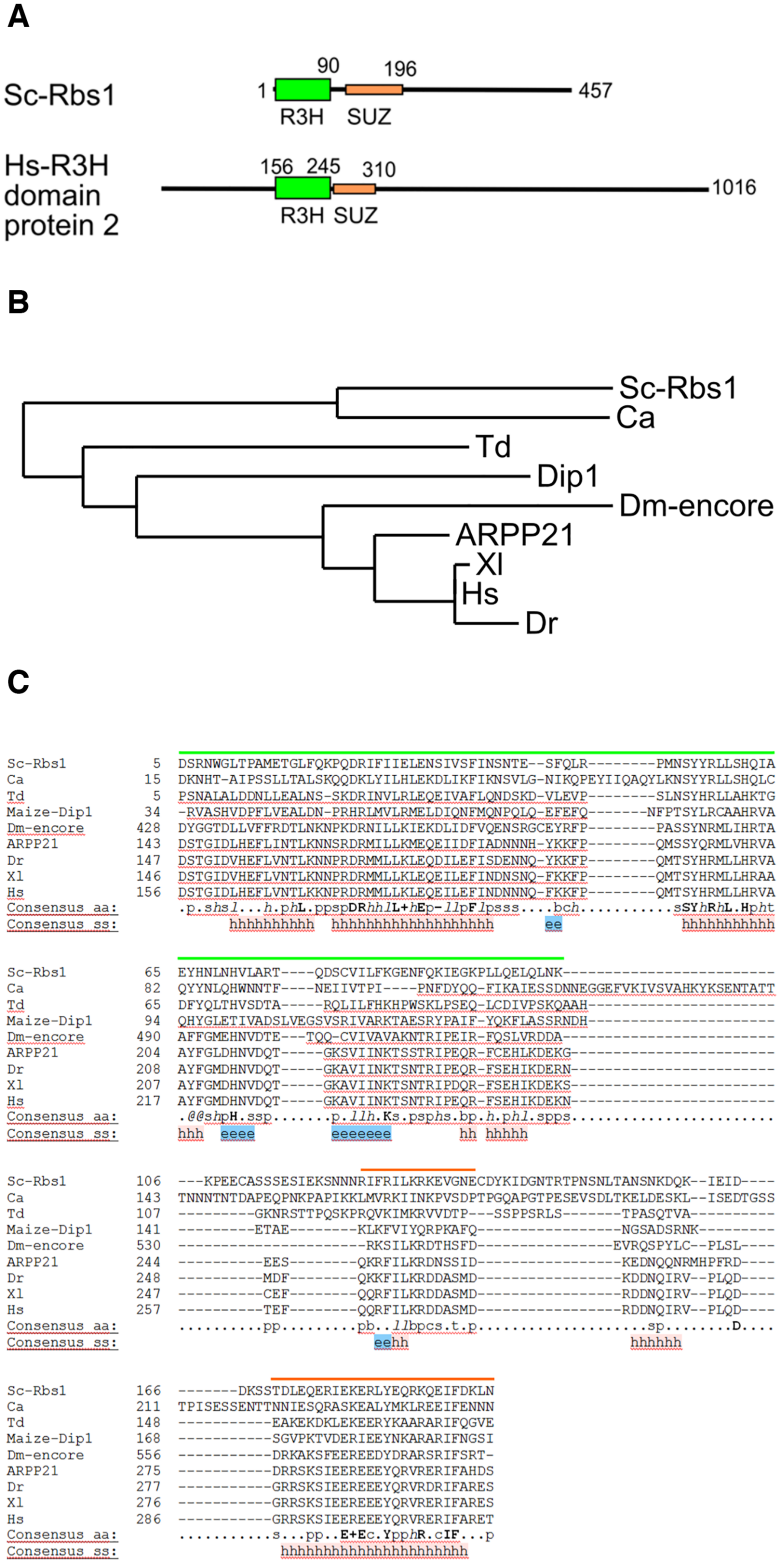
Evolutionary conservation of potential Rbs1 orthologues. (**A**) Schematic diagram of the domain composition of Sc-Rbs1 and human R3H domain-containing protein 2. Green rectangles indicate R3H domains. Orange rectangles indicate the SUZ domain. The taller rectangles for R3H indicate a more structured nature of this domain over less structured SUZ (see Figure 1A). (**B**) Phylogenetic tree of selected potential orthologues of Rbs1 generated with phylogeny.fr (25,26). (**C**) Sequence alignment of the R3H-SUZ region of selected Rbs1 homologues (output from Promals3D (24)). Green lines indicate the R3H domain. Orange lines indicate the SUZ domain. The following symbols apply, with sequence IDs in parentheses: Sc-Rbs1, *Saccharomyces cerevisiae* Rbs1; Ca, hypothetical protein (*Candida albicans*, KHC63810.1); Td, putative R3H domain protein (*Taphrina deformans*, CCG82637.1); Maize Dip1 (DIP1 *Zea mays*, AAZ73119.1); Dm-encore, encore protein (*Drosophila melanogaster*, NP_995992.1); ARPP21, (*H. hapiens*, XP_016861070), Hs, R3H domain-containing protein 2 (*H. sapiens*, XP_011536342.1); Dr, R3H domain-containing protein 2 (*Danio rerio*, XP_021333011.1); Xl, R3H domain-containing protein 2-like (*Xenopus leavis*, XP_018105556.1). Consensus amino acid residues in bold are conserved in all sequences: l, aliphatic; @, aromatic; h, hydrophobic; o, alcohol; p, polar residues; t, tiny; s, small; b, bulky residues; +, positively charged; –, negatively charged; c, charged. Predicted/determined secondary structure (consensus ss): h, helices; e, strands

One interesting issue is whether equivalents of Rbs1 play similar roles in other organisms, particularly animals. In order to address this issue, we performed a preliminary search for metazoan Rbs1 homologues using BLAST. Interestingly, this initial analysis revealed not only the conservation of the R3H domain (aa 5–90 in Rbs1) but also in a region located downstream to the R3H domain (aa 124–195 in Rbs1). Further analysis of this region revealed the presence of motifs that are characteristic of the SUZ domain (Figure 10A and C). The SUZ domain was identified in *C. elegans* Szy-20 protein, where it was shown to bind RNAs suggesting that SZY-20 might function in the localization, stability, or translation of RNA at the centrosome to locally regulate expression of one or more proteins (47). *S. cerevisiae* Rbs1 region, comprising the R3H and SUZ domains, is 48% identical and 92% similar between Rbs1 and human R3H domain-containing protein 2 (sequence ID: XP_011536342.1). No sequence conservation is found in the C-terminal portion of the proteins, outside the RH3-SUZ region, and no easily identified domains are found there. Therefore, the R3H-SUZ portion is likely the main functional module of these proteins.

Next, we searched the literature for other proteins that would contain an R3H-SUZ domains combination. Alike yeast Rbs1, ARPP21 protein from mouse possess adjacent N-proximal R3H and SUZ domains and a large, unstructured C terminus. ARPP21 is mRNA binding protein which recognizes uridine-rich sequences with high specificity for 3′UTRs. Furthermore, ARPP21 physically interacts with the components of the translation initiation factor eIF4F suggesting a role of ARPP21 in translation (48).

We also found two other proteins comprising R3H-SUZ domains—DIP1 from maize and encore from *Drosophila melanogaster*, both of which were partially characterized. DIP1 interacts with DBF1 protein, which is involved in the response to abiotic stress (49). DIP1-DBF1 binding controls expression of the aba-responsive *rab17* gene during stress. Encore is involved in the post-transcriptional regulation of Grk (gurken) mRNA. Importantly, spatiotemporal control of gurken mRNA translation is required for establishing the embryonic body axes. Encore was also implicated in the RNA turnover of *bam*, another gene that controls differentiation in *Drosophila* (50,51). Both DIP and encore proteins are involved in RNA metabolism, supporting their functional relationship with Rbs1.

For more in-depth analysis of the R3H-SUZ proteins from fungi, plants and animals we performed PSI-BLAST searches which are described in detail in Materials and Methods section. Alignment of the R3H-SUZ regions from the proteins found using PSI-BLAST and their phylogenetic tree are shown in Supplementary Figure S2 and, in a simplified form in Figure 10B and C. Several conclusions can be drawn from this sequence analysis. Rbs1 homologues with R3H-SUZ domain combination are widely present in fungi, plants and animals. The R3H-SUZ domain region of Rbs1 homologues from vertebrates is very well conserved. In the proteins from fungi, plants and animals the SUZ domain shows high sequence conservation with a motif EERXXXYXXXRX+IF (where ‘+’ stands for positively charged residue) located in a predicted α-helix (Figure 10C and Supplementary Figure S2). This implies that the R3H-SUZ portion is likely the main functional module of these proteins and the identified sequences are functional equivalents of Rbs1 in other eukaryotes. This possibility is further supported by the fact that human R3H domain protein 2 (R3HDM2) found in our search was identified as an RNA-binding protein (17). Apart from R3HDM2, DIP1 and encore, all the retrieved sequences correspond to uncharacterized proteins. Therefore, our analysis identified a group of widespread R3H-SUZ proteins which are likely involved in mRNA regulation.

## DISCUSSION

The present study found novel levels of regulation that reveal cooperation between Rbs1 and Rpb10 proteins in assembly of the RNA Pol III complex. Rbs1 is a poly(A) mRNA-binding protein that interacts with Upf1 helicase and controls Rpb10 expression at the level of mRNA. Both mRNA binding by Rbs1 and the participation of this protein in Pol III assembly depend on the functional R3H domain. Rbs1 is involved in a more general regulatory mechanism that controls mRNA that might be conserved in not only in fungi but also in plants and metazoans, which was suggested by the identification of Rbs1 orthologues in other organisms, including humans.

**Figure 11.**
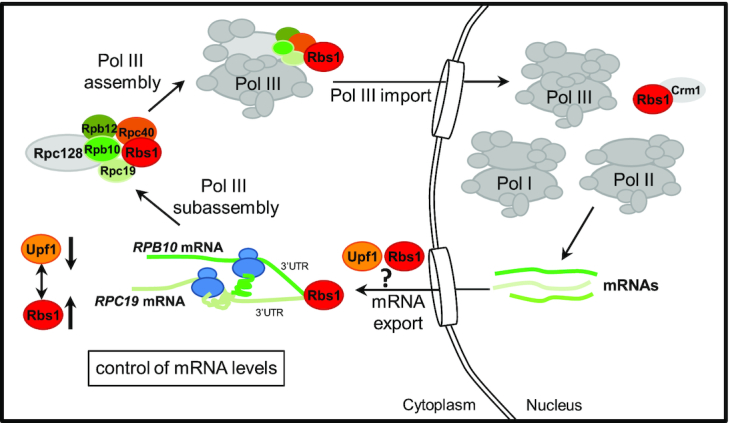
Early steps in Pol III biogenesis in the yeast *Saccharomyces cerevisiae* that connect the control of Rpb10 expression and its role in assembly of the Pol III complex through a regulatory loop that involves Rbs1 protein. See explanation in text.

The previously characterized *rpc128*-*1007* mutant that is defective in assembly of the Pol III complex was a successful genetic tool in the present study. A clear growth phenotype of this mutant previously allowed the selection of Rpb10 and Rbs1 as overdose suppressors, and both proteins have been shown to play a role in Pol III biogenesis (11). Rpb10 is a part of the stable subcomplex (Rpc128–Rpc40–Rpc19–Rpb12–Rpb10) that is probably formed during the initial step of Pol III assembly (7,8), Figure 11. Noteworthy, mutations of the conserved motif of Rpb10 lead to a complete depletion of the largest Pol I subunit, Rpa190 suggesting dissociation of the Pol I complex from the mutant enzyme which is not properly assembled and supporting a role of Rpb10 in assembly of Pol I (10). Possibly, Rpb10 is involved in coordination of the levels of mature Pol I and Pol III enzymes at the step of their assembly.

Rbs1 protein physically interacts with subunits of the Pol III complex and stimulates their mutual interactions when overproduced. Additionally, Rbs1 interacts with the Crm1 exportin and shuttles between the cytoplasm and nucleus. This led us to propose a previous model in which Rbs1 binds to the Pol III complex or subcomplex and facilitates its translocation to the nucleus (11).

Here, we used the *rpc128*-*1007* mutant to examine the role of Rbs1 domains in Pol III assembly. The genetic results clearly showed functional significance of the R3H domain and no involvement of the prionogenic domain (Figure 1). This implies a contribution of the RNA-binding potential of Rbs1 to Pol III assembly. The involvement of the R3H domain in the interaction between Rbs1 and the Pol III complex and Crm1 exportin was unlikely. Therefore, we explored indirect effects of Rbs1 on Pol III assembly through an influence on Rpb10 expression.

Different approaches that we applied herein strongly support a regulatory link between Rbs1 and Rpb10. First, the molecular study found an increase in *RPB10* mRNA levels upon Rbs1 overproduction with the active R3H domain (Figure 2) and this led to elevated level of Rpb10 protein (Figure 6E). Second, the genetic analysis revealed a role for the 3′ regulatory region of *RPB10* mRNA in suppressing the Pol III assembly defect by Rbs1 (Figure 3) and this result was confirmed by CRAC analysis showing direct binding to this part of *RPB10* mRNA (Figure 9C). These findings suggest that the control of Rpb10 expression by Rbs1 and the 3′ regulatory region in *RPB10* mRNA is crucial for regulation of Pol III assembly.

Perhaps, Rbs1 stimulates translation of Rpb10 protein by the interaction of the R3H domain with the regulatory region in *RPB10* mRNA. Tempting speculation is that Rbs1 recruits mRNA encoding another Pol III subunit which is simultaneously synthesized by polysomes in close physical proximity and interacts with Rpb10 while being translated. On the basis of CRAC analysis, such simultaneous model could be proposed for co-translational assembly of Rpb10 and Rpc19 subunits. Alternatively, Rbs1 binds and recruits another mature Pol III subunit to the 3′UTR of *RPB10* mRNA which undergoes translation. The candidate subunits are Rpc19, Rpc40 and Rpb5 identified previously among proteins that physically interact with Rbs1 (11).

Co-translational assembly, reported so far for several multisubunit complexes (ex. SAGA or fatty acid synthase FAS) has been postulated as a general principle in yeast and mammalian cells (52–54). Our novel insight supports the hypothesis that co-translational assembly linked with the regulation of Rpb10 abundance has a central role in the biogenesis of Pol III complex.

According to the current model (Figure 11), Rbs1, by interaction with mRNA, brings together Rpb10, Rpc19 and possibly other subunits during translation process. This emerging picture can now explain a role of Rbs1 in assembly of polymerase complex postulated previously (11). As discussed below, this model deliberates join function of Rbs1 and Upf1 in RNA metabolism including control of mRNA export from the nucleus.

A functional link of Rbs1 with Upf1, the main player in the NMD surveillance mechanism has been provided here by several lines of evidence. The physical interaction between Rbs1 and Upf1 that was identified in previous studies (11,23) was confirmed by co-immunoprecipitation and further investigated (Figure 5). We have shown that Rbs1-Upf1 interaction is stronger in the absence of the RNA, but this effect is independent of RNA binding by Rbs1. This implies that Upf1 is able to bind either RNA or Rbs1. This may be attributable to the overlap of the two binding interfaces on the surface of Upf1. A tempting speculation is that this may serve as a mechanism that makes the regulatory mechanism more robust. An increase in Rbs1 expression would promote the protection of a target mRNA by Rbs1 and at the same time block the ability of Upf1 to bind and destabilize this mRNA. RNase treatment liberates Upf1 from RNA thereby making it accessible for interaction with Rbs1 produced in excess. The exact molecular details of this mechanism will require further detailed biochemical and structural studies.

The physical interaction results in the present study indicate functional cooperation between Upf1 and Rbs1. Here, we have shown that inactivation of the Upf1 gene increased steady-state levels of *RPB10* mRNA and corrected the Pol III assembly defect in the *rpc128*-*1007* mutant (Figure 6). These interesting findings demonstrate the involvement of Upf1 in Pol III assembly. The levels of others mRNA that were controlled by Upf1 in 3′ UTR-dependent NMD decay were finely tuned by Rbs1 (Figure 7). Rbs1 had no effect on the precursor of *RPL28* mRNA, the main target of 5′ UTR NMD. Only transcripts that are subjected to degradation by 3′ UTR NMD are likely protected by Rbs1 overproduction in yeast cells. Our Rbs1 CRAC analysis clearly demonstrates binding of the 3′ UTRs of mRNA transcriptome-wide. Although we were unable to identify genome-wide correlation between Rbs1 targets and NMD targets defined by Celik and colleagues (55), we propose that Rbs1 functions, at least in part, by opposing NMD-mediated degradation.

In higher eukaryotes, UPF1 mainly acts on 3′ UTRs of mRNAs and is estimated to regulate 5–10% of human genes. In addition to detecting aberrant transcripts, NMD has also been shown to target a broad range of mRNAs under normal conditions (56). Much of this regulation is achieved by specific *cis*-acting sequence elements and *trans-*acting RNA-binding proteins that control NMD. Specific sequence elements that are found near termination codons play a role in protecting genuine long 3′ UTRs in many transcripts by binding specific factors (57). To date, two proteins, PTBP1 and hnRLP L, have been shown to play a role in shielding normal transcripts from NMD (56,57). They share many functional and structural properties but recognize different sequences in 3′ UTRs.

Another intriguing aspect of Rbs1 function is suggested by its physical interaction with Yra1 and Nop3/Nlp3 proteins that are involved in controlling mRNA export. A role for Upf1 in mRNA export should also be considered. According to a recent report, UPF1 protein in *Drosophila* shuttles between the cytoplasm and nucleus in a Crm1-dependent fashion. UPF1 is required for the release of mRNAs from their transcription sites in the nucleus and plays an important role in their export from the nucleus (58). The rapid export and resulting evacuation of mRNA from exosomes in the nucleus secure their stability (59). Possibly the greater efficiency of mRNA export contributes to the role of Rbs1 in protecting mRNAs from degradation.

Clearly, Rbs1 binding to poly(A) mRNA is mediated by the R3H domain. One example of a poly(A)-binding protein that contains the R3H domain is the human poly(A)-specific RNase PARN. Remarkably, deletion of the R3H domain prevented PARN from binding to the poly(A) substrate and dramatically reduced cleavage activity (28).

In the light of the above, a natural question is, whether mechanisms of the global control of RNA metabolism similar to the one described in this work operate in other organisms, in particular in metazoans. Our analysis of Rbs1 homologs revealed a presence of proteins with the combination of R3H and SUZ domains in many species of fungi, plants and animals. In these sequences, the only conserved regions are the R3H domain and the SUZ domain with latter containing highly conserved sequence motifs. This implies that the R3H-SUZ region is the main functional module of these proteins. Both R3H and SUZ domains in isolation have been implicated in RNA binding and regulation (34,47). Potential Rbs1 orthologue, ARPP21 that contains the combination of the two domains and long C-terminal unstructured region is involved in interaction with 3′UTRs in mRNA and translation regulation (48). Such an involvement would be very interesting when considering the connection of Rbs1with co-translational assembly of Pol III and other RNA polymerases.

## Supplementary Material

gkaa1069_Supplemental_File
