# Supplementary material for: The expression of Rpb10, a small subunit common to RNA polymerases, is modulated by the R3H domain-containing Rbs1 protein and the Upf1 helicase
Source: Nucleic Acids Res. 2020 Nov 24;48(21):12252–68. doi: 10.1093/nar/gkaa1069 (PMC7708074; doi:10.1093/nar/gkaa1069)
Supplement: gkaa1069_Supplemental_File [file gkaa1069_supplemental_file.pdf]

SUPPLEMENTARY DATA

SUPPLEMENTARY MATERIALS AND METHODS

Yeast strains, media and growth conditions.
BY4741, MB159-4D and MJ15-9C yeast strains used in this study were described previously (1, 2). Heterozygous diploid *RPB10*/*rpb10*Δ (BY4743 MATa/MATα *ura3*Δ*0/ura3*Δ*0 leu2*Δ*0/leu2*Δ*0 his3*Δ*1/his3*Δ*1 met15*Δ*0/MET15 LYS2/lys2*Δ*0 YOR210w/YOR210w::kanMX*) and the deletion mutant *upf1*Δ (BY4741 MATa *ura3*Δ*0 leu2*Δ*0 his3*Δ*1 met15*Δ*0 YMR080c::kanMX*) were obtained from Euroscarf. *RPB10*/*rpb10*Δ was transformed with plasmids containing wild type or mutated *RPB10* alleles and subjected to meiosis. Generated *rpb10*Δ haploids expressing *RPB10* alleles from the plasmids were subsequently crossed with the *rpc128-1007* mutant (MJ15-9C MATa *SUP11 ade2-1 ura3-1 lys2-1 leu2-3,112 his3*) resulting in *rpc128-1007 rpb10*Δ[*RPB10*] or *rpc128-1007 rpb10*Δ[*RPB10* Δ3'154]. The strain Upf1-TAP (MATa *ade2-1 his3-11,15 leu2-3,112 trp1-1 ura3-52 UPF1 TAP HIS3*) kindly supported by A. Dziembowski, was crossed with *rbs1*Δ (BY4742 *MATα his3*Δ*1 leu2*Δ*0 lys2*Δ*0 ura3*Δ*0 YDL189w::kanMX4*) or Rbs1-Myc (*MATα SUP11 ade2-1 ura3-1 lys2-1 leu2-3,112 his3 YDL189w::13Myc::KanMX6*) (1) that resulted Upf1-TAP *rbs1*Δ or Upf1-TAP Rbs1-Myc strains.
The *rpc128-1007 upf1*Δ strain was obtained by transformation of *upf1*Δ deletion cassette, which was amplified by PCR from the genomic DNA of BY4741 *upf1*Δ by using specific Upf1For (5'-AGAAGGAAGGGCAGCAAGAC-3') and Upf1Rev (5'-GCGCTCATTTCACGGTTGAG-3') primers. Transformants were selected on YPD medium supplemented with geneticin (200 μg/ml) and the replacement of *UPF1* with the kanamycin cassette was confirmed by PCR.
The RBS1-HTP strain was obtained by transformation of the appropriate sequence encoding the HTP tag, which was amplified by PCR using the pBS1539-HIS6-TEV-ProtA plasmid as DNA template and specific primers, RBSHTP_F (5'-CTAGGGATACTGATTCGGTAGAGATGAAATTTGATAAATTAAACATTTAGGAGCA CCATCACCATCACC-3') and RBS1HTP_R (5'-TCTATAAACCGTTACGTAATTTTTTCGCTATGTATAGTTCACTCCTCACGTACGAC TCACTATAGGG-3'). Transformants were selected on SC-ura medium. The presence of RBS1-HTP-encoding sequence was confirmed by western blot method with the PAP antibody.
As required, the YPD medium was supplemented with geneticin (200 μg/ml). Sporulation medium contained 0.05% glucose, 0.1% yeast extract and 1% potassium acetate. LB medium (1% Bacto tryptone, 0.5% yeast extract, 1% NaCl, pH 7.5) was used for growing *E. coli* strains. As required, the LB medium was supplemented with ampicillin (60 μg/ml). Solid media contained 2% agar. For analysis of the phenotypes, yeast strains grown on SC-ura, SC-leu or SC-ura-leu medium were replica-plated on YPD plates and incubated for 3 days at the desired temperature. Yeast cells were transformed with plasmids by using the lithium acetate method (3).

Plasmids
YEp181-*RBS1*, called here [*RBS1*], multicopy plasmid (*LEU2*, 2μ) containing the *RBS1* gene (1). YEp181-*RBS1* R3H, called here [*RBS1* R3H], the conserved Arg57 aa and His61 aa, located within the R3H domain, were changed to alanine using primers Mut_rev (5'-ATAGAAG**AGC**ATAATATGAATTCATCGGTC-3') and Mut_for (5'-C**CGC**CCAAATAGCTGAGTACCACAA-3'). YEp181-*RBS1*-Myc and YEp181-*RBS1* R3H-Myc, called here [*RBS1*-Myc] and [*RBS1* R3H-Myc] contain the wild type or mutated version

of *RBS1* tagged with Myc epitope at the 3' termini. They were obtained by transformation of BY4741 *rbs1*Δ [*RBS1*] or [*RBS1* R3H] with the His3MX6 cassette PCR amplified from pFA6a-13Myc-His3MX6 plasmid using primers RBS1_F2 (5'-TACTGATTCGGTAGAGATGAAATTTGATAAATTAAACATTCGGATCCCCGGGTTAATTAA-3') and RBS1_R1 (5'-ACTAGAATTTCTATAAACCGTTACGTAATTTTTTCGCTATGTATAGTTCACTCCTCGAATTCGAGCTCGTTTAAAC-3') (4). Deletion of the respective restriction fragments from the YEp181-*RBS1* resulted in derivative plasmids, called here [*RBS1* ΔC1] and [*RBS1* ΔC2], containing deletion of fragments of *RBS1* encoding 231-332 aa and 231-457 aa, respectively.
Fragments containing 211 bp upstream and 154 bp, 231 bp or 253 bp downstream of the *RPB10* ORF were amplified by PCR the [*RPB10*] plasmid DNA by using RPBF (5'-GATGGCTACTACACTGGAAG-3') and RPBR (5'-CCTACAGTATGCAGAGACAC-3'), 231RpbR (5'-ACTTCCTTTATCGTCTTGAAGAGT-3') or 253RpbR (5'-TCGCACGATGTAACATCTACA-3') primers, respectively. PCR products were introduced to pDrive vector (Qiagen), cut by restriction enzyme EcoRI and cloned into EcoRI site of centromeric plasmid pRS316 (*URA3 CEN6*) resulting in plasmids called here [*RPB10* Δ5'Δ3' 154], [*RPB10* Δ5'Δ3' 231] or [*RPB10* Δ5'Δ3' 253]. Both [*RPB10* Δ3' 154] and [*RPB10* Δ5'] plasmids were constructed in the same way. [*RPB10* Δ3' 154] plasmid containing 663 bp upstream and 154 bp downstream of the *RPB10* ORF was amplified by PCR from the pFL44L-RPB10a plasmid (5) by using MGMF (5'-AGACAGCAGCATGCATCG-3') and RPBR (described above) primers. [*RPB10* Δ5'] plasmid containing 211 bp upstream and 647 bp downsteam of the *RPB10* ORF was amplified by PCR from the pMJ18 plasmid (1) with RPBF (described above) and MGMR (5'-ATTGACCTCCACAGCTG-3') primers.
HA-epitope sequence fused at N-terminus of *RPB10* gene with a 5' and 3' regulatory regions, 250 bp and 390 bp, respectively (synthetized by Syngen biotech company) in pEX-A128 vector was cloned to pRS316 in NotI and EcoRI sites, called here [HA-*RPB10*].

RNA isolation and northern hybridization
Total RNA was isolated by heating and freezing the cells in the presence of SDS and phenol as described previously (6). Samples containing 20 μg of total RNA were denaturated in a glyoxal reaction mixture at 55°C for 1 h (7) and were resolved by electrophoresis in 1.2 % agarose gel in 1×BPTE buffer (10 mM PIPES, 30 mM Bis-Tris, 1 mM EDTA pH 8.0). RNA samples were transferred into a Hybond-N+ membrane (Amersham) with 10×SSC using the TurboBlotter downward capillary transfer system (Schleicher & Schull) and crosslinked by UV radiation (0.14 J/cm^2). The blot was prehybridized for 3 h at 65°C in buffer containing 7% SDS, 0.5 M Na_2HPO_4 pH 7.4, 1 mM EDTA, 1% BSA, and hybridized with *RPB10*, *ACT1* and *SCR1* probes. DNA probes were amplified by PCR using oligonucleotides listed in the Supplementary Table 1 and were labeled with [α-^{32}P]-dATP by random priming using the HexaLabel DNA labeling kit (Fermentas). Hybridization was carried out overnight at 65°C in the same buffer as prehybridization. Filters were washed three times (15 min each) with 2×SSC, 0.1% SDS at 65°C. Hybridization signals were exposed to a phosphorimager screen. RNA was quantified using the PhosphorImager STORM 820 (Molecular Dynamics). Band intensities were quantified using the MultiGauge v3.0 software (Fujifilm).

Supplementary Table 1. Oligonucleotides for northern hybridization

**Target**	**Forward**	**Reverse**
RPB10N	ATCAGCACTCCGATGGCTAC	ACAAGTCTCTTATATCGCACGA
ACT1N	TTCCCATCTATCGTCGGTAG	GTGGTGGAGAAAGAGTAACC

SCR1N	TCTGGTGGGATGGGATACGT	TCAGGACACACTCCATCCCC

cDNA synthesis and reverse transcription-quantitative PCR (RT-qPCR)
100 nanograms of RNA was used for cDNA synthesis using a QuantiTect reverse transcriptase kit (Qiagen). cDNA for each sample was performed according to the manufacturer's instruction. RT-qPCR reactions contained 1 µL of cDNA template, 300 nM primer pairs and 5 µL of RT PCR Mix SYBR (A&A Biotechnology). Quantitative PCR was performed on a Roche LightCycler 480 System using a 5-min incubation at 95°C, followed by 40 cycles of 30 s at 95°C, 20 s at 60°C, and 20 s at 72°C (with a plate read after each cycle). A melting curve analysis was performed for each sample after PCR amplification to ensure that a single product with the expected melting curve characteristics was obtained. Each sample was loaded in triplicate. Each plate contained cDNA dilutions for the standard curve, a non-reverse transcriptase control, and a no template control. PCR efficiencies were between 90% and 100%. Data were processed in LightCycler 480 Software and then analyzed in Excel (Microsoft). Data are expressed in arbitrary units calculated from standard curve where the highest cDNA concentration was set to 1. The primer sequences are listed in the Supplementary Table 2.

Supplementary Table 2. Oligonucleotides for RT-qPCR

**Target**	**Forward**	**Reverse**
RPB10	TGTTGGTGACAAGTGGGAAA	GATCGACGTGGGTTAGAATCA
PGA1	ACCACTCGGCTAGGTATCCC	CGTTCAATACGTGTGGTGCC
MSH1	ACTTGGTCGTGGTTCAAGCA	CGTTGCCTCCGTTCGAAGTA
SPO16	ATTTCGCAGCTTAACGAGGA	GGAACTCCAGGGCTTTTCTG
CNN1	AAGCATTGGAATGGATCAGG	TCCTCTTTATCGTCGCTTGG
pre-RPL28	CCATCTCACTGTTGAGACGG	CTCAGTTTGCGATGGAAGAG
RPL28	TCACGTCTCAGCCGGTAAAG	ATGTTGACCACCGGCCATAC
ACT1	CATGTTCCCAGGTATTGCCGA	GTCAAAGAAGCCAAGATAGA
SCR1	GAGAATTCTGGCCGAGGAACAAA	TCTGCCCAGGACAAATTTACGA

Protein extraction and western blot analysis
The protein extraction method was described earlier (8). Protein extracts were separated by 6% or 10% sodium dodecyl sulphate-polyacrylamide gel electrophoresis (SDS-PAGE). After electrophoresis proteins were transferred onto the nitrocellulose membrane (Milipore), which was then blocked in TBST (10mM Tris, 150 mM NaCl, 0.05% Tween 20) containing 5% fat-free dry milk for 30 min and subsequently incubated with the appropriate antibody: mouse monoclonal antibodies 9E10 anti-Myc (Roche) at a 1:2,000 dilution for 1 h, anti-HA (Covance) at a 1:5,000 dilution for 2h, anti-Pgk1 (Abcam) at a 1:20,000 dilution for 1 h, anti-Nab2 (gift from Torben Heick Jensen) at a 1:1,000,000 dilution for 1 h and rabbit polyclonal antibodies anti-Rbs1 (Gramsh) at a 1:1,000 dilution for 1h. Blots with TAP-tagged Upf1 protein were incubated with anti-PAP antibody at a 1:3,000 dilution. PAP is a peroxidase anti-peroxidase antibody which does not necessitate the use of a secondary antibody. Then membrane was incubated for 1 h with a secondary anti-mouse or anti-rabbit antibody coupled to horseradish

peroxidase (DAKO) at a dilution of 1:5,000. The signal on the membrane was visualized by chemiluminescence using the ECL detection kit (Bio-Rad).

CRAC analysis

Samples were processed as previously described (PMID: 30718516, PMID: 32585128) with modifications. Cells were lysed in TNMC100 (50 mM Tris-HCl pH 7.5, 150 mM NaCl, 0.1% NP-40, 5 mM $MgCl_2$, 10 mM $CaCl_2$, 20U of DNase RQ1 and a protease-inhibitor cocktail (1 tablet / 50 mL) with zirconia beads in a 50 mL conical. The cells were lysed with five one-minute pulses, with cooling on ice in between. The supernatant was spun for 20 minutes at 21,000g. The cleared lysate was incubated with the IgG Sepharose for two hours at 4°C, with nutating. Subsequently, the beads were washed two times with TMN600 (50 mM Tris-HCl pH 7.5, 600 mM NaCl, 0.1% NP-40, 1.5 mM $MgCl_2$) and two times TMN100 (50 mM Tris-HCl pH 7.5, 100 mM NaCl, 0.1% NP-40, 5 mM $MgCl_2$). For RNA digestion beads were resuspended in 600 μL TMN100 containing 0.04U of RNase-IT and samples were incubated for 10 minutes at 23 °C 1000 rpm to fragment protein-bound RNA. RNase digestion was slowed down by incubation on ice for 3 min.

The beads were washed three times TMN100 and resuspended in 600 μL TMN100 containing 5 μL HaloTEV (Promega). Protein: RNA complexes were eluted by incubation with HaloTEV for 2h at 18°C with shaking. The supernatant was separated and adjusted for nickel affinity purification with the addition of 400 mg guanidine hydrochloride, 45 μl NaCl (3M) and 7 μL imidazole (1 M) and added to 50 μL of washed nickel beads (Qiagen).

Following 2h incubation, the nickel beads were washed three times with WBI (6.0 M guanidine hydrochloride, 50 mM Tris-HCl pH 7.5, 300 mM NaCl, 0.1% NP-40, 10 mM imidazole, 1.5 mM $MgCl_2$), three times with C buffer (50mM Tris-HCl pH 7.5, 50 mM NaCl, 1.5 mM MgCl2, 0.1% IGEPAL CA-630) and twice with 50 mM Bis-Tris pH 6.5 and transferred to a spin column. Subsequent reactions (80 μl total volume for each) were performed in the columns, and afterward washed once with WBI and three times with PNK buffer:

1. Dephosphorylation (70 mM Bis-Tris pH 6.5, 10 $MgCl_2$, 40 U T4 PNK (NEB), 2 U TSAP (Promega); 37°C 30 min)

2. 3' linker ligation (70 mM Tris-HCl pH 7.5, $MgCl_2$, 12.5% PEG8000, 40U T4 RNA Ligase II truncated K227Q, 80U RNasIN, 80 pmol preadenylated 3' miRCat-33 linker (IDT); 16°C overnight).

3. 5' end phosphorylation and radiolabeling (70 mM Tris-HCl pH 7.5, $MgCl_2$, 40 U T4 PNK (NEB), 80U RNasIN, 40 μCi ^{32}P-γATP; 37°C for 45 min, with addition of 100 nmol of ATP after 30 min).

4. 5' linker ligation (70 mM Tris-HCl pH 7.5, $MgCl_2$, 12.5% PEG8000, 40 U T4 RNA ligase I (NEB), 80 U RNasIN, linker, 200 pmol 5' linker, 1 mM ATP; 16°C for 3h and 25°C for 2h).

The beads were washed twice with WBI and three times with C buffer. Protein: RNA complexes were eluted 100 μL of elution buffer (50 mM Tris-HCl pH 7.5, 500 mM NaCl, 0.1% Triton X-100, 6M guanidine hydrochloride and 300 mM imidazole), supplemented 3 μL GlycoBlue and ethanol precipitated overnight (9 volumes of ethanol). RNPs were pelleted at 21000g for 20 minutes, washed twice 80% acetone, rehydrated in 12 μL water. 4 μL 4X NuPAGE sample loading buffer supplemented with 8% β-mercaptoethanol. The sample was denatured by incubation at 65°C for 10 minutes, and run on a 4%–12% Bis-tris NuPAGE gel at 130 V. The protein: RNA complexes were transferred to Hybond-C nitrocellulose membranes with NuPAGE MOPS transfer buffer for 2 h at 100V.

Labelled RNA was detected by autoradiography. The appropriate region was excised from the membrane and treated with 0.2 μg/μL Proteinase K (50 mM Tris-HCl pH 7.5, 50 mM NaCl,

0.1% IGEPAL CA-630, 1% SDS, 5 mM EDTA; 2 hr 55°C with shaking) in a 500 µL reaction. The RNA component was isolated with a standard phenol:chloroform extraction followed by ethanol precipitation with 2 µL of GlycoBlue. The RNA was reverse transcribed using Superscript IV and the miRCat-33 RT oligo (IDT) for 15 min at 50°C in a 20µL reaction. Subsequently 2 µL ExoI (NEB) were added, incubated 30 min at 37°C and heat inactivated 20 min at 80°C. The resulting cDNA was amplified by PCR in 50 µL reactions using Phusion (Thermo) (2 µL template, 19 cycles) PCR reactions were combined, purified and concentrated using AmpureXP beads, and resolved on a 3% Metaphore agarose gel. A region corresponding to 150 to 250 bp was excised from the gel and extracted using the Zumoclean Gel DNA Recovery Kit (Zymo Research). Libraries were measured with Qbit and sequenced using Illumina NextSeq 550 with 150bp single-end reads.

Quantification and statistical analysis of CRAC data

Pre-processing and data alignment

Illumina sequencing data were demultiplexed using in-line barcodes and in this form were submitted to GEO. First quality control step was performed using FastQC software (http://www.bioinformatics.babraham.ac.uk/projects/fastqc/) considering specificity of CRAC data. Raw reads were trimmed with flexbar v3.4.0 (Dodt et al., 2012) with parameters –q TAIL –qf i1.8 –qt 20 to remove bases with QC<20. Subsequently reads were collapsed to remove PCR duplicates using FASTX-collapser v0.0.14 (http://hannonlab.cshl.edu/fastx_toolkit/) then inline barcodes were removed using pyBarcodeFilter.py script from pyCRAC package v3.0 (9). The 3' adapter were removed using flexbar v3.4.0 (10) with parameters –as TGGAATTCTCGGGTGCCAAGGC -u 3 –m 17 –n 16 –bt RIGHT.

All datasets were aligned to the yeast genome using Novoalign v2.07.00 (http://www.novocraft.com) with parameter –r random and saved in novo format to calculate classes of bound RNAs and BigWig files for visual inspection in IGV genome browser. Second quality control step was performed using pyReadCounters script (pyCRAC package) which calculates overlaps between aligned cDNAs and yeast genomic features. The 1 nt resolution BigWig files were generated using bamCoverage v3.1.3 script from deepTools package (11). Sam file operations were performed using SAMtools v1.9 (12).

Additionally all datasets were aligned to yeast transcriptome with parameter using STAR aligner v.2.7.3a (13) and only unambiguously mapped reads were used to generate binding profiles.

Downstream analyses were performed using python 3.6.10 Jupiter notebooks, python libraries (pandas v0.19.2, numpy v1.16.0, scipy v1.2.0, matplotlib v2.2.3) and in-house scripts submitted available s an update of gwide toolkit v0.5.27 (https://github.com/tturowski/gwide) (14).

Identifying Rbs1 enriched genes

To compare CRAC data and RNA-seq all reads mapping to mRNAs were normalized to reads per million (RPM) and converted to log2 RPM. For each transcript p-value was calculated using a two-sided T-test. High confidence Rbs1 targets were selected using following criteria: p-value <0.01, ratio between Rbs1 binding and RNA-seq >1.5 and >128 uniquely mapped RPM.

Rbs1 metagene representation

For metagene analysis data were processed independently for each replicate. Reads mapped to each transcript were summed up to 1 and fraction of reads was used further. This excluded risk that the obtained profile is biased by the most abundant transcripts, as each transcript has a value of 1. To combine transcripts of different length, for metagene representation fraction of

reads was binned as follows: 5’UTR 10 bins, CDS 100 bins and 3’UTR 10 bins. Average fraction of reads was plotted for each bin.

Binding to mature mRNA

Reads mapped to each transcript were summed up to 1 and fraction of reads was used further. Fraction of reads mapping to each feature was counted. Boxplots present 2nd and 3rd quartile, line marks median and whiskers range between 5th and 95th percentile.

REFERENCES

1. Cieśla,M., Makała,E., Płonka,M., Bazan,R., Gewartowski,K., Dziembowski,A. and Boguta,M. (2015) Rbs1, a new protein implicated in RNA polymerase III biogenesis in yeast Saccharomyces cerevisiae. *Mol. Cell. Biol.*, **35**, 1169–1181.

2. Ciesla,M., Skowronek,E. and Boguta,M. (2018) Function of TFIIIC, RNA polymerase III initiation factor, in activation and repression of tRNA gene transcription. *Nucleic Acids Res.*, **46**, 9444–9455.

3. Chen,D.C., Yang,B.C. and Kuo,T.T. (1992) One-step transformation of yeast in stationary phase. *Curr. Genet.*, **21**, 83–84.

4. Longtine,M.S., McKenzie,A., Demarini,D.J., Shah,N.G., Wach,A., Brachat,A., Philippsen,P. and Pringle,J.R. (1998) Additional modules for versatile and economical PCR-based gene deletion and modification in Saccharomyces cerevisiae. *Yeast*, **14**, 953–961.

5. Lalo,D., Carles,C., Sentenac,A. and Thuriaux,P. (1993) Interactions between three common subunits of yeast RNA polymerases I and III. *Proc. Natl. Acad. Sci. U.S.A.*, **90**, 5524–5528.

6. Schmitt,M.E., Brown,T.A. and Trumpower,B.L. (1990) A rapid and simple method for preparation of RNA from Saccharomyces cerevisiae. *Nucleic Acids Res.*, **18**, 3091–3092.

7. Sambrook,J. and Russell,D.W. (2001) Molecular cloning: a laboratory manual Cold Spring Harbor Laboratory Press, Cold Spring Harbor, N.Y.

8. Towpik,J., Graczyk,D., Gajda,A., Lefebvre,O. and Boguta,M. (2008) Derepression of RNA polymerase III transcription by phosphorylation and nuclear export of its negative regulator, Maf1. *J. Biol. Chem.*, **283**, 17168–17174.

9. Webb,S., Hector,R.D., Kudla,G. and Granneman,S. (2014) PAR-CLIP data indicate that Nrd1-Nab3-dependent transcription termination regulates expression of hundreds of protein coding genes in yeast. *Genome Biol.*, **15**, R8.

10. Dodt,M., Roehr,J.T., Ahmed,R. and Dieterich,C. (2012) FLEXBAR-Flexible Barcode and Adapter Processing for Next-Generation Sequencing Platforms. *Biology (Basel)*, **1**, 895–905.

11. Ramírez,F., Ryan,D.P., Grüning,B., Bhardwaj,V., Kilpert,F., Richter,A.S., Heyne,S., Dündar,F. and Manke,T. (2016) deepTools2: a next generation web server for deep-sequencing data analysis. *Nucleic Acids Res*, **44**, W160–W165.

12. Li,H., Handsaker,B., Wysoker,A., Fennell,T., Ruan,J., Homer,N., Marth,G., Abecasis,G., Durbin,R. and Subgroup,1000 Genome Project Data Processing (2009) The Sequence Alignment/Map format and SAMtools. *Bioinformatics*, **25**, 2078–2079.

13. Dobin,A., Davis,C.A., Schlesinger,F., Drenkow,J., Zaleski,C., Jha,S., Batut,P., Chaisson,M. and Gingeras,T.R. (2013) STAR: ultrafast universal RNA-seq aligner. *Bioinformatics*, **29**, 15–21.

14. Turowski,T.W., Leśniewska,E., Delan-Forino,C., Sayou,C., Boguta,M. and Tollervey,D. (2016) Global analysis of transcriptionally engaged yeast RNA polymerase III reveals extended tRNA transcripts. *Genome Res.*, **26**, 933–944.

SUPPLEMENTARY FIGURES

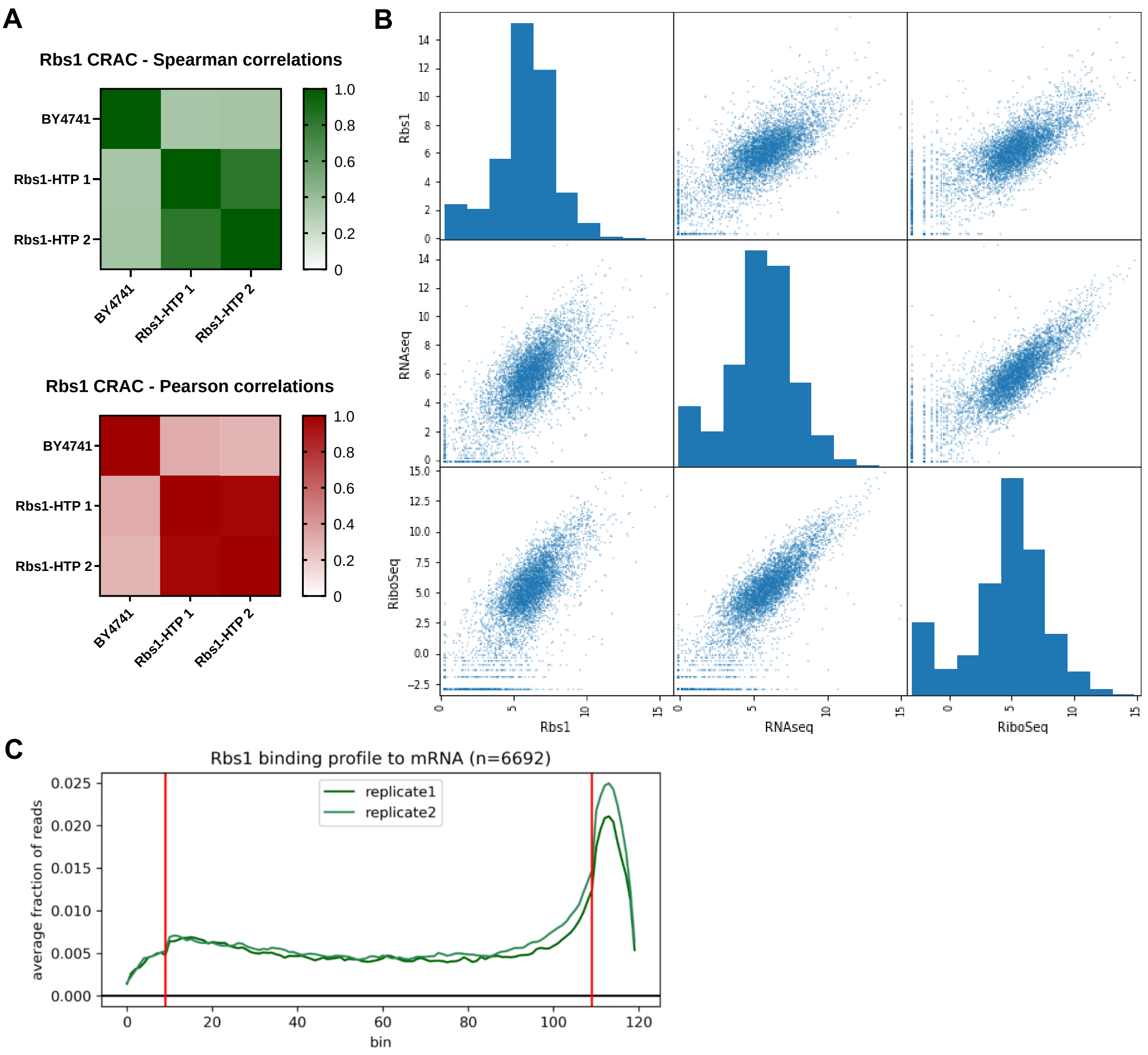

Supplementary Figure S1. Statistical analysis of CRAC data for Rbs1 over all mature mRNAs. (**A**) Spearman and Pearson correlations between datasets. (**B**) Scatter plots comparing the Rbs1 RNA binding to RNA-seq and Ribo-seq data. Reads uniquely mapping to mRNAs are presented (log2 RPM). (**C**) Metagene representation of read density over all mature mRNAs (n=6692). Data were separated into 120 bins: 10 for the 5' UTR, 100 for the CDS and 10 for the 3' UTR. Horizontal lines indicate were CDS starts and stops. Metagene analysis performed for mRNA containing at least 100 reads in Rbs1 CRAC data (n=6692).

A

XP_010757305.1	hypothetical protein PADG_02291 [Paracoccidioides brasiliensis Pb18]
RMJ22032.1	R3H domain protein [Aspergillus sp. HF37]
QGA13340.1	hypothetical protein EYB26_000988 [Talaromyces marneffei]
OQE15951.1	hypothetical protein PENSTE_c026G04122 [Penicillium steckii]
XP_002585083.1	predicted protein [Uncinocarpus reesii 1704]
PGH19146.1	hypothetical protein AJ80_04226 [Polytolypa hystricis UAMH7299]
XP_033404196.1	uncharacterized protein GIQ15_05635 [Arthroderma uncinatum]
KAF2399143.1	hypothetical protein EJ06DRAFT_72354 [Trichodelitschia bisporula]
KAF1939774.1	hypothetical protein EJ02DRAFT_407933 [Clathrospora elynae]
ELQ33482.1	hypothetical protein OOU_Y34scaffold00936g9 [Pyricularia oryzae Y34]
TQS37745.1	hypothetical protein Golomagni_01771 [Golovinomyces magnicellulatus]
SZF03220.1	unnamed protein product [Blumeria graminis f. sp. hordei]
KFZ15736.1	hypothetical protein V502_05427 [Pseudogymnoascus sp. VKM F-4520 (FW-2644)]
XP_016645040.1	R3H domain-containing protein [Scedosporium apiospermum]
KFH40507.1	R3H domain-containing protein-like protein [Acremonium chrysogenum ATCC 11550]
CCE34041.1	uncharacterized protein CPUR_07972 [Claviceps purpurea 20.1]
CEJ80227.1	hypothetical protein VHEMI00424 [Torrubiella hemipterigena]
KAB5577609.1	hypothetical protein GE09DRAFT_1213834 [Coniochaeta sp. 2T2.1]
XP_014168719.1	r3h domain protein [Grosmannia clavigera kw1407]
PSR74187.1	hypothetical protein BD289DRAFT_226619 [Coniella lustricola]
VBB75180.1	Putative protein of unknown function [Podospora comata]
XP_009848197.1	hypothetical protein NEUTE1DRAFT_144342 [Neurospora tetrasperma FGSC 2508]
KIH88101.1	r3h domain protein [Sporothrix brasiliensis 5110]
XP_030994999.1	uncharacterized protein E0L32_006261 [Phialemoniopsis curvata]
KXJ91167.1	hypothetical protein Micbo1qcDRAFT_119375 [Microdochium bolleyi]
KKA30932.1	hypothetical protein TD95_004645 [Thielaviopsis punctulata]
AJV06700.1	Rbs1p [Saccharomyces cerevisiae YJM1418]
GCF00085.1	hypothetical protein ZYGM_002314 [Zygosaccharomyces mellis]
OVA05989.1	Single-stranded nucleic acid binding R3H [Macleaya cordata]
GEU59184.1	R3H domain-containing protein 1-like [Tanacetum cinerariifolium]
OWM87646.1	hypothetical protein CDL15_Pgr022759 [Punica granatum]
XP_008808576.1	uncharacterized protein LOC103720587 [Phoenix dactylifera]
MQL81330.1	hypothetical protein Taro_013800, partial [Colocasia esculenta]
XP_020699935.1	R3H domain-containing protein 1 [Dendrobium catenatum]
MQM11394.1	hypothetical protein Taro_044302 [Colocasia esculenta]
KMZ62657.1	Single-stranded nucleic acid binding R3H protein [Zostera marina]
PKA67145.1	hypothetical protein AXF42_Ash004637 [Apostasia shenzhenica]
XP_009386294.1	R3H domain-containing protein 2 [Musa acuminata subsp. malaccensis]
XP_020254643.1	uncharacterized protein LOC109831676 isoform X1 [Asparagus officinalis]
XP_020254456.1	cAMP-regulated phosphoprotein 21-like isoform X2 [Asparagus officinalis]
OAY67162.1	R3H domain-containing protein 1 [Ananas comosus]
KAF3787699.1	R3H domain-containing protein 1 [Nymphaea thermarum]
XP_023741289.1	R3H domain-containing protein 2 isoform X2 [Lactuca sativa]
KAE9465814.1	hypothetical protein C3L33_02274, partial [Rhododendron williamsianum]
KAE8697742.1	Single-stranded nucleic acid binding R3H protein isoform 2 [Hibiscus syriacus]
XP_006402961.1	R3H domain-containing protein 2 [Eutrema salsugineum]
XP_010552904.1	uncharacterized protein LOC104823164 [Tarenaya hassleriana]
XP_022966122.1	uncharacterized protein LOC111465900 isoform X1 [Cucurbita maxima]
GER51925.1	single-stranded nucleic acid binding R3H protein [Striga asiatica]
XP_020114255.1	uncharacterized protein LOC109728293 isoform X1 [Ananas comosus]
ERM99095.1	hypothetical protein AMTR_s00101p00122520 [Amborella trichopoda]
XP_006654401.1	uncharacterized protein LOC102704805 isoform X1 [Oryza brachyantha]
KAF3341400.1	R3H domain-containing protein 1 [Carex littledalei]
XP_034191896.1	R3H domain-containing protein 1-like isoform X3 [Osmia lignaria]
XP_024218457.1	cAMP-regulated phosphoprotein 21 isoform X2 [Halyomorpha halys]
GFG30713.1	hypothetical protein Cfor_07482, partial [Coptotermes formosanus]
XP_015115923.1	R3H domain-containing protein 1 isoform X3 [Diachasma alloeum]
XP_029828169.1	R3H domain-containing protein 2 [Ixodes scapularis]
XP_022254084.1	cAMP-regulated phosphoprotein 21-like isoform X2 [Limulus polyphemus]
XP_015903829.1	cAMP-regulated phosphoprotein 21-like [Parasteatoda tepidariorum]
XP_023222768.1	R3H domain-containing protein 2-like [Centruroides sculpturatus]
XP_002423078.1	R3H domain-containing protein, putative [Pediculus humanus corporis]
XP_019865105.1	cAMP-regulated phosphoprotein 21 [Aethina tumida]
XP_022901769.1	cAMP-regulated phosphoprotein 21 isoform X2 [Onthophagus taurus]
XP_017778862.1	cAMP-regulated phosphoprotein 21 isoform X4 [Nicrophorus vespilloides]
VEN60483.1	unnamed protein product [Callosobruchus maculatus]
KAF2898948.1	hypothetical protein ILUMI_07232 [Ignelater luminosus]
XP_030766515.1	cAMP-regulated phosphoprotein 21 isoform X3 [Sitophilus oryzae]
XP_018019452.1	cAMP-regulated phosphoprotein 21-like [Hyalella azteca]
XP_014672054.1	cAMP-regulated phosphoprotein 21-like [Priapulus caudatus]
XP_014675087.1	cAMP-regulated phosphoprotein 21-like, partial [Priapulus caudatus]
XP_030385838.1	protein encore [Scaptodrosophila lebanonensis]
XP_029725418.1	protein encore isoform X2 [Aedes albopictus]
XP_032599088.1	protein encore isoform X4 [Drosophila grimshawi]
KNC31935.1	Protein encore, partial [Lucilia cuprina]
XP_020714208.1	protein encore isoform X4 [Ceratitis capitata]
XP_023246188.1	R3H domain-containing protein 1 [Copidosoma floridanum]
XP_023329389.1	cAMP-regulated phosphoprotein 21-like [Eurytemora affinis]
XP_029201356.1	cAMP-regulated phosphoprotein 21-like [Acropora millepora]
XP_021368844.1	cAMP-regulated phosphoprotein 21-like [Mizuhopecten yessoensis].
XP_034305772.1	cAMP-regulated phosphoprotein 21 isoform X6 [Crassostrea gigas]
XP_013815153.1	R3H domain-containing protein 1 isoform X3 [Apteryx mantelli mantelli]
XP_016861070	ARPP-21/cAMP-regulated phosphoprotein 21 isoform X3 [Homo sapiens]

B

```
                                        6
Rbs1                              -----SRNWGLTPAMETGLFQKPQDRIF-------------IIELENSIVSFINSNT--ESFQLRP----
Ca                                ------KNHTAIPSSLLTALSKQQDKLY-------------ILHLEKDLIKFIKNSVL-GNIKQPEYIIQ
XP_010757305.1                    EQVLHSFPNEPDEKLLEAMIS-PKDRLL-------------ILQLEEKIIAFIKDSAE-QSLELPP----
RMJ22032.1                        DRAIHGFPHEPDEKLLEAMES-PKDRLL-------------ILQLEDMVRHFIQKSKE-QSLELPP----
QGA13340.1                        GKSLNGYALEPDEKLLEAMGS-PKDRLF-------------LLQLEEMIRDFIQDSKE-QSLELPP----
OQE15951.1                        GSGVQGLPQEPDEKLLEAMKT-PKDRLL-------------ILQLEEKITNFIKDSEE-QSLELPP----
XP_002585083.1                    ERTLHGFPNEPDEKLLEAMRS-PKDRLL-------------ILQLEEKIICFIKNPSE-QSLELPP----
PGH19146.1                        AQLLHGFPKDPDEKLLEAMNS-PKDRLL-------------LLQLEEKIVAFIKDSR-----QLPP----
XP_033404196.1                    ERNLHGFPSEPDEKLMEAMSS-PKDRLL-------------LLQLEEKIVSFIKNSSE-GSLELPP----
KAF2399143.1                      -------VPTPDEKLVEALES-PRDRVY-------------VLKIEQDVIDFLKYSKD-SQLPLPQ----
KAF1939774.1                      -------PAIPDEKLLEALQS-PRDRLF-------------VVKIEQDFIDFIKDSRE-SEYSLPN----
ELQ33482.1                        -----FYSQNPDEKLLEAMKS-SKDRIF-------------LLRLEKEVIEFVQDSKE-PFMDLPP----
TQS37745.1                        -----FYRQTPDEKLLEALDS-VKDRIF-------------LLRLEQEVIEFVRSSKE-PFIDLPP----
SZF03220.1                        -----FYRQTPDDKLLEALDS-VKDRIF-------------LLRLEQDVIEFVRSSKE-PFIDLPP----
KFZ15736.1                        -----FYKHTPDEKLIEALES-PKDRIF-------------LLRLEQDVIEFVKDSKE-PFIDLPP----
XP_016645040.1                    -----IYRKAPDEKLLEAMQS-PKDRIF-------------LLRLEKDVIDFVRDSKE-PYMDLPP----
KFH40507.1                        -----IYRHAPDEKLIEALAT-PKDRIY-------------LLRLEKEVINFVQNSGE-PYMDLPP----
CCE34041.1                        -----IYRQAPDEKLLLALTS-PRDRFF-------------LLRLEKDVIDFVQDSKE-PYMDLPP----
CEJ80227.1                        -----IYRQAPDDKLLDALAT-PRDRYF-------------LLRLEKDVMDFVQNSKE-PYMDLPP----
KAB5577609.1                      ---NMLYRQAPDEKLLEAIQS-PKDRLF-------------LLRLEAQVINFVQDSKE-PFMDLPP----
XP_014168719.1                    -------GQSPDEKLLDAMQT-PKDRLF-------------LLRLESDVVNFVQNSKE-PFMDLPP----
PSR74187.1                        ------YGQNPDEKLLEAMSS-QKDRLF-------------LLKLEETVIDFVQDSKE-PFMDLPP----
VBB75180.1                        ----GFLSQHPDEKLLEAMQS-HKDRIF-------------LLRLEQQVIEFVQDSKE-PFMDLPP----
XP_009848197.1                    --NGGFLSQHPDEKLLEAMRS-EKDRFS-------------LLKMEQQVIEFVENSKE-PFMDFPP----
KIH88101.1                        -------GQNPDEKLLDAMAS-QKNRVF-------------LLRLESEVVDFVQNSKE-PFMDLPP----
XP_030994999.1                    -----GYRQNPDDKLLEAMRS-PKDRLF-------------LLRLEKDVIEFVQDSKE-PFMDLPP----
KXJ91167.1                        -----VYGQNPDEKLIEAMKS-PKDRIF-------------LLRLEQDVINFVQSSKE-PYMDLPP----
KKA30932.1                        -----IYQQAPDDKLLEALGS-TKDCQF-------------LLRLEVDVISFVQESKE-PYMDLPP----
Td                                -----SNALALDDNLLEALNS-SKDRIN-------------VLRLEQEIVAFLQNDSK-DVLEVPS----
AJV06700.1                        -----SRNWGLTPAMETGLFQKPQDRIF-------------IIELENSIVSFINSNT--ESFQLRP----
GCF00085.1                        -----FVGLGLTPAMMTALFHKPHDRQF-------------VIELENSITSFINSNA--ESYELRP----
OVA05989.1                        -----DATNQVDQFLREALQN-PRERLS-------------ILRMEQDVEKFIRDPTQ-QQLEFQQ----
GEU59184.1                        -----LLDDSVDQFLREALQN-PRERLS-------------VLRMEQDVEKFIRDPSQ-QQMEFQQ----
OWM87646.1                        -----DAIDQVDQFLREALQN-PRERLS-------------ILRMEQDMEKFIRDPTQ-QQFEFQQ----
XP_008808576.1                    -----AALLQVDQFLLEALEN-PRHRLT-------------VLRMELDIQRFMQSSDL-HQFEFQH----
MQL81330.1                        ----KEGLDQVDPFLVEALEN-PRLRLTVYEL-----ALILVLRMELDIQKFMQNHGL-HQFEFQQ----
XP_020699935.1                    -------FSQVDPFLVEALDN-PRHRLT-------------VLRMELEIQQFMQNPDQ-YQFEFQP----
MQM11394.1                        -----EAPPQVDPFLVEALEN-PRHRLT-------------VLRMELDIQRFMQIPDQ-YQFEFQH----
KMZ62657.1                        -------VSKIDPLVFEALEN-PRHRLT-------------VLRMELDVQSFMRNSSQ-HQFEFQH----
PKA67145.1                        -----GKLLQIDPFLVEALEN-PRHRLT-------------VLRMELDIQRFMQSSDQ-YQFEFQH----
XP_009386294.1                    -----ELLSQVDPFLLEVLEN-PRHRLT-------------VLRMELDIQRFMQNPYH-QHFEFQP----
XP_020254643.1                    -------ESHVDPFLVEALEN-PRHRLT-------------VLRMELDIQKFVRNPNQ-HQFEFHH----
XP_020254456.1                    -------SSQVDQFLVEAIEN-PRHRLT-------------VLRMELDIQKFMQNPDQ-HQFEFQH----
OAY67162.1                        -----MGLNPVDPFLVEALEN-PRHRLT-------------VLRMELDIQKFMQNPNL-QEFEFQH----
KAF3787699.1                      -----DAYPDVDPFLKEAIQN-PRHRLT-------------VLRMELDIQRFMQNTEQ-HQFEFQH----
XP_023741289.1                    -----YATMAVDPFLVEALQN-PRHRLS-------------ILRMELDIQKFIQNIEQ-HQFEFQH----
KAE9465814.1                      -----DGVVAVDPFLVEALQN-PCHRLT-------------ILRMELDVQKFLKNSDQ-QQFEFQH----
KAE8697742.1                      -----DKESSVDPFLVEALQN-PRHRLT-------------ILRMELDIQKFLQHSDQ-QQFEFQH----
XP_006402961.1                    ----------VDPFLVEALQN-PRHRLT-------------ILRMELDVQRFLQNLEQ-QQFEFQH----
XP_010552904.1                    -----EKETMVDPFLVEALQN-PRHRLT-------------ILRMELEVQKFLRNTEQ-DQFEFQH----
XP_022966122.1                    -----EKLSMVDPFLVEALQN-PRHRLT-------------ILRMELDIQRFLENQEQ-QVFEFQH----
GER51925.1                        -----AYVSEVDPFLVEALQN-PRHRLTIHTVKHISPFPISVLRMELDVQNFMQLPDL-HQFEFPH----
XP_020114255.1                    -----GMPVPVDPFLVEALGN-PRHRVTEVVALC-IPIPTAVLRMEMDIQRFMKNPDL-YQFEFPH----
ERM99095.1                        ---GHIQHLQVDRFLLEALQN-PRHRLT-------------VLRMELDIQMLMKNPEQ-QQLELPH----
XP_006654401.1                    -----RVTSHVDPFLVEALDN-PRHRLM-------------VLRMEMDIQKFMQNPQL-HEFEFQH----
Dip1                              -----RVASHVDPFLVEALDN-PRHRLM-------------VLRMELDIQNFMQNPQL-QEFEFQN----
KAF3341400.1                      ----------VDPFLVELLEN-PRYRLI-------------VLRMELDIQKFMQNPEL-QEFEFQH----
XP_034191896.1                    -----STGIDLEHFIAETINRNQKDRTV-------------LLKIEKDLIEFARDKQK-VCHKFPN----
XP_024218457.1                    -----GNQVDLHQFIVDTLNRNHKDRML-------------LLKIENELVNLANDPKR-TMHKFAQ----
GFG30713.1                        -----STGVDLQQFIMDTLNRNQKDRIL-------------LLKIEQELVSLAKDNKR-NHYKFPQ----
XP_015115923.1                    -----STGIDLEQFIAVTINRNQKDRSV-------------LLKIEKELIEFAKDRQK-VCHKFPN----
XP_029828169.1                    -----STGVDLEQFIVDTLHKNHKDRVM-------------MLKIEQDLLSLVRDNKR-QSLRFAQ----
XP_022254084.1                    -----STGINLQQFIIETLHKNQKDRLT-------------MLKIEQEIISLIKDSKR-QSFKFPH----
XP_015903829.1                    -----SSGIDLQQFIIDTLHKNQKDRLM-------------VLKIEQELINLIKDNKK-HSIKFPQ----
XP_023222768.1                    -----STGIDIQQFIIDTLHKNQKDRMM-------------LLKIEQELINLVKDGKR-QSHKFPQ----
XP_002423078.1                    -------GIDLNQFIADTLNRNYKDRML-------------LLQIEQDLISLIRDKR--SHQKFPL----
XP_019865105.1                    -----TTGVDLEVFIPETLNRNAKDRAL-------------MLRIEQELVTLAKDKNQ-THYKFPP----
XP_022901769.1                    -----TTGVNLVKFIQETLNKNMKDRQL-------------LLKIEQELLTLCECNEK-THFKFPA----
XP_017778862.1                    -----TTGVDLEKFIPETLNRNAKDRAL-------------MLRIEEELTQLAKDSGK-THYKFPP----
VEN60483.1                        -----TTGIDLEQFIPETLNRNSKDRAL-------------MLRIEQELVNLAKDRCK-THYKFPP----
KAF2898948.1                      -----TTGVDLEQFIAETLNRNGKDRAL-------------MLRIEQELVSLAKDKIR-THYKFPP----
XP_030766515.1                    -----TTGVNLEQFIPETLNRNPKDRAL-------------MLRIEEELILLANDKFQ-THYKFPP----
XP_018019452.1                    -----SSGVDLLQFIIQTLHKHQKDRAV-------------LLKIERELINLVKDNKR-SHHKFQQ----
XP_014672054.1                    -----STGIDLEEFISMTLHKNPNDRRV-------------LLKLEQDMTSLVKDTKR-QLMKFQP----
XP_014675087.1                    -----SAGVDLEEFFMMTLHRNLSDRRM-------------LIKLEEQMSGLVKDSKR-HMMKFNP----
XP_030385838.1                    -----YGGTDLLMFFRDTLNKNPKDKHI-------------LLKIEKDLIEFVQEKGRGCEFRFPP----
XP_029725418.1                    -----QSGVNLIQFFKETLNKNFKDRNM-------------LMKIEKELLALAMDRTR-SQIKFPP----
Dm_encore                         -----YGGTDLLVFFRDTLNKNPKDRNI-------------LLKIEKDLIDFVQENSRGCEYRFPP----
XP_032599088.1                    -----YGGTDLLTFFRDTLNKNPKDRNI-------------LMKIEKDLMDFIQDKGRDCEFRFPP----
KNC31935.1                        -----YGGTDLLSFFKETLNKNVKDRQF-------------LLKVEKDLIEFVQEGNR-GEIRFPQ----
XP_020714208.1                    -----HGGTDLFSFFKETLNKHPKDRHF-------------LLKVEKDLTEFVQEKSR-GELRFPP----
XP_023246188.1                    -----STGTDLELFIAETINRNQKDRQH-------------LLKIERELIDFAKDKHK-ASHRFIS----
XP_023329389.1                    -----STGIDLTQFIISTLNKNTKDRML-------------MLKLETDMTALVKDPKK-GHQKFPQ----
XP_029201356.1                    -----STGTDLHDFIIKTLKN-PRDRKF-------------LMKLEQDFLNFIREPL--MFLHYPP----
XP_021368844.1                    -----STGINLEDFIRKTMNKTPKDRNM-------------LLKLETDLTNFINEPKH-HYLKFPQ----
XP_034305772.1                    -----STGINLEDFIHKTINKSPKDRNM-------------LLKLETDLINFINEPKH-HYLKFPQ----
XP_013815153.1                    -----STGIDLHEFLVNTLKNNPRDRMM-------------LLKLEQEILDFIGNNEV-PRKKFPP----
XP_016861070 - ARPP21            -----STGIDLHEFLINTLKNNSRDRMI-------------LLKMEQEIIDFIADNNN-HYKKFPQ----
Hs/Xl/Dr                          -----STGIDVHEFLVNTLKNNPRDRMM-------------LLKLEQEILEFINDNSN-QFKKFPQ----
Consensus aa:                     ...........p.bh.phh.p..+cRhh.............lL+hE.-l.pFhpsspp...hch......
Consensus ss:                                hhhhhhhhhh hhhhhh             hhhhhhhhhhhhh       eee
```

```
                                   52
Rbs1                       ----MNSYYRLLSHQIAEYHNLNHVLAR--------TQDS-CVILFKGENFQKIEGKP-----------L
Ca                         AQYLKNSYYRLLSHQLCQYYNLQHWNNT---------FNE--IIVTP--IPNFDYQQF------------
XP_010757305.1             ----CNAFGRLLAHKLGDYYHLTHFVDN--------NVTS--VRLHRTPWCRLPTPLS------------
RMJ22032.1                 ----SNAFGRLLAHKLGDYYHLTHFVDN--------NVTS--VRLHRTPFCRLPTPLS------------
QGA13340.1                 ----SNAFGRLLAHKLGDYYHLTHFVDN--------NVTS--VRLHRTPFCRLPTPLS------------
OQE15951.1                 ----SNAFGRLLAHKLGDYYHLTHFVDN--------NVTS--VRLHRTPFCRIPTPLS------------
XP_002585083.1             ----CNAFGRLLAHKLGDYYHLTHFVDN--------NVTS--VRLHRTPWCRLPTPLS------------
PGH19146.1                 ----CNAFGRLLAHKLGDYYHLTHFVDN--------NVTS--VRLHRTPWSRLPTPLS------------
XP_033404196.1             ----CNSFGRLLAHKLGDYYHLTHFVDN--------NVTS--VRLHRTPWSRLPTPLT------------
KAF2399143.1               ----TNAFYRMLAHRLADYYGLEHTVEH--------SGSGAAVVITKTGNSRMPAPLT------------
KAF1939774.1               ----CNTFYRMLAHRLADYYLLGHVVDT--------TMTG--VKITRTPYCRIPPPVS------------
ELQ33482.1                 ----CNSFCRLLTHKLADYYHMTHSFEA--------VAGA--VRIYRTPFCRVPPSLQ------------
TQS37745.1                 ----CNSFCRMITHKLADYYHLTHQVDA--------VAGA--VRIFRTPFCRLPPSLT------------
SZF03220.1                 ----CNSFCRMLTHKLADYYHMTHQVDA--------VAGA--VRIFRTPFCRLPPSLT------------
KFZ15736.1                 ----CNSFCRMLTHKLADYYHMTHQFDA--------VAGS--VRIFRTPFCRLPQSLT------------
XP_016645040.1             ----CNSFCRMLTHKLADYYHMTHSYEP--------SIGA--VRIFRTPFCRVPQSLA------------
KFH40507.1                 ----SNSFCRMLAHKLADYYHMTHSFEP--------NIGV--VRIFRTPFCRVPTSLA------------
CCE34041.1                 ----SNSFCRMLTHKLADYYHMTHSYEP--------HVGS--VRIFRTPFCRVPPSLA------------
CEJ80227.1                 ----SNSFCRMLTHKLADYYHMTHSYEP--------HIGS--VRIFRTPFCRVPPSLA------------
KAB5577609.1               ----CNSFCRMLTHKLADYYHMTHSFEA--------VAGA--VRIYRTPFCRVPAPLS------------
XP_014168719.1             ----SNSFCRMLTHKLADYYHMTHSFEA--------VAGS--VRIFRTPFCRVPPPLA------------
PSR74187.1                 ----SNSFCRMLTHKLADYYHMTHSFEA--------AAGA--VRIFRTPFCRVPPSLA------------
VBB75180.1                 ----SNSFCRMLMHKLADYYHMTHSFES--------QAGA--VRIFRTPFCRIPPSLS------------
XP_009848197.1             ----NNTFWRMLTHKLADYYHMTHSYEA--------VSGA--VRIYRTPFCRIPPPLS------------
KIH88101.1                 ----SNSFCRMLTHKLADYYHMTHSFEA--------VAGS--VRIYRTPFCRVPPPLA------------
XP_030994999.1             ----CNSFCRMLTHKLADYYHMTHSFEA--------VAGA--VRIYRTPFCRVPPSLA------------
KXJ91167.1                 ----NNSFCRMLTHKLADYYHMTHSFEA--------VQGA--VRIYRTPFCRVPPSLA------------
KKA30932.1                 ----SNSFFRMLTHKLADYYHMTHSYEK--------DNGPGFVRIFRTPFCRVPPSLA------------
Td                         ----LNSYHRLLAHKTGDFYQLTHVSDT--------ARQL--ILFHKHPWSKLPSEQL------------
AJV06700.1                 ----MNSYYRLLSHQIAEYHNLNHVLAR--------TQDS-CVILFKGENFQKIEGKP-----------L
GCF00085.1                 ----MNSYYRLLSHQVAEYHNLKHTLAR--------ARDN--CVIIFKGDGFENMQGKPFLQNLHPMGLV
OVA05989.1                 ---LPTSYLRLAAHRVAQHYCLQSMVVLDNNLPD-GSGSR--IIVRKTSECRFPPIRL------------
GEU59184.1                 ---LPTSYLRLAAHRVAQHYSLQSMVLLDNTLPD-GSGSR--IIVRKTSECRVPLIRL------------
OWM87646.1                 ---LPTSYLRLAAHRVAQHYSLQSMVLLDSNSLD-GSGSR--IIVRKTSECRLPAIRL------------
XP_008808576.1             ---LPTSYLRCAAHRVAQHYGLQTMVADSTLDG---LNGR--IIARKTSESRFPAVFL------------
MQL81330.1                 ---FPSSYLRRAAHRVAQHYGLQTMVLDNLLDG----IGR--IVVRKTPDSRFPAICL------------
XP_020699935.1             ---FPTSYLRCAAHRVAQHYGLQTMSVDNITDG---SGSR--IVVRKTSESRYPLTCL------------
MQM11394.1                 ---LSSSYLRCAAHRVAQHYGLLTIVLDNNLDG----RGR--IIVRKTPESRYPVVCL------------
KMZ62657.1                 ---YPTSYLRCAAHRVAQHYGLQTMVLDITSDG----SGK--IVAWKTPESRFPDVCL------------
PKA67145.1                 ---FSTSYLRCAAHRVAQHYGLQTLVVDTTGDG---SCSR--IAARKTPESRYPSILL------------
XP_009386294.1             ---LPTSYLRCAAHRVAQHYGLQTMALDNVVDG---LGSR--VVARKTPQSKYPVVCL------------
XP_020254643.1             ---LPTSYLRCAAHRVAQHYGLQTFSLDNTIDSLGTLGSK--ILAIKTPGSKFPTVCL------------
XP_020254456.1             ---FPTSYLRCAAHRVAQHYGLQTSALDYTVDG---PGSK--IVARKTPESKFPAICL------------
OAY67162.1                 ---FPTSYLRCAAHRIAQHYGLETTVADSILDC---SSSR--IIARKTSVSKIRSISL------------
KAF3787699.1               ---FPTSYLRLAAHRVAQHYGLQSMVVDADGYG---IGGK--IVVKKTPESKFPSTCL------------
XP_023741289.1             ---FPTSYLRLAAHRVAQHYGLQTMVLDNFTDG---QGTR--ILVKKLPESKYPSICL------------
KAE9465814.1               ---FPTSYLRLAAHRVAQHYGLQTMVQDNNVLD--GLGTT--IVVKKLADSRYPATCL------------
KAE8697742.1               ---FPTSYLRLAAHRVAQHYGLITMVHDSSLGG---VGSR--IVAKRAVEFRCPAVRL------------
XP_006402961.1             ---FPTSYLRLAAHRVANHYGLATAVQDSGVDG---NENI--ILVTKTTESRFPAVRL------------
XP_010552904.1             ---YPTSYLRLAAHRVAQHYGLVTMAMDGGLDG---SGNR--ILVRKTAEIRYPSVCL------------
XP_022966122.1             ---FPTSYLRLAAHRVAQHYGLQTMVQDNGIDG----QGR--ILVRNTGEARFPPICL------------
GER51925.1                 ---FPTSYLRLAAHRVAQHYGLQTIVQD-VVDG---QGIR--ITVIKKPESKFPVVCL------------
XP_020114255.1             ---FATSYLRRCAHRVAQHYGLQTMSLDNTTDG---SGSR--VMARKTLDSKFPAICL------------
ERM99095.1                 ---FPTSYLRLAAHRVALHYGLQSMASDNTADG---SGSR--IIVRKTPESRYPSICL------------
XP_006654401.1             ---FPTSYLRCAAHRVAQHYGLETTVADSLVDG---SVSK--IVAKKTPESKFPVIAL------------
Dip1                       ---FPTSYLRCAAHRVAQHYGLETIVADSLVEG---SVSR--IVARKTAESRYPAIFY------------
KAF3341400.1               ---LPTSYLRCAAHRVAQHYGLETTAADSIVNS---SLVN-KVIARKTPNSKFPSLLL------------
XP_034191896.1             ----MSSYNRMLVHRVAAYFGMEHNVDQ--------SGSS--VIVTRTKNMRIPNTRF------------
XP_024218457.1             ----MSSYQRMLVHRVAAYFSMEHNVDT--------SGVA--VIVNTTKSTRIPDTRF------------
GFG30713.1                 ----MSSYQRMLVHRVAAYFGMDHNVDQ--------TGNA--VIVNRTKNTRLPDTKF------------
XP_015115923.1             ----MSSYNRMLVHRVAAYFGMEHNVDQ--------SGLS--VIVTRTKNMRIPDTRF------------
XP_029828169.1             ----MSSYHRMLVHRVAAYFGLDHNVDQ--------AGTA--VIVNKTRNTRLPEMRF------------
XP_022254084.1             ----MSSYHRMLVHRVAAYFGLDHNVDQ--------NGTA--VIVNKTKNTRLPETKF------------
XP_015903829.1             ----MSSYNRMLVHRVAAFFGLDHYVDG--------NGTS--VIVNKTKTSRIPDTRF------------
XP_023222768.1             ----MSSYNRMLVHRVAAFFRLDHYVDQ--------NGTA--VIVNKTKNTRLPDIKF------------
XP_002423078.1             ----MSSYQRMLVHRVAAYFGMDHNVDQ--------TGHC--VVVSVTPSTRLPEIRF------------
XP_019865105.1             ----MSSYQRMLVHRCAAYFGMDHNIES--------SGKC--VVVNKTKATRIPDVPF------------
XP_022901769.1             ----MSSYQRMLVHRVAAYFGMDHNIEP--------SGKS--VVVNKTRNTRIPEDRF------------
XP_017778862.1             ----MSSYQRMLVHRCAAYFGMDHNIEP--------SGKC--VVVNKTKNTRIPDVEF------------
VEN60483.1                 ----MSSYQRMLVHRCAAYFGMDHNIES--------SGGK-SVIVNKTKNTRIPETEF------------
KAF2898948.1               ----MSSYQRMLVHRCAAYFGMDHNIES--------SGKC--VVVNKTKCTRIPDVAF------------
XP_030766515.1             ----MSSYHRMLVHRCAAYFGMDHNIEQ--------SGKC--VVVNKTKNTRIPDVKF------------
XP_018019452.1             ----MSSYHRMLVHRCAAYFGLEHNVDQ--------AGTA--VIVNKTKNTRLPEVRF------------
XP_014672054.1             ----MSSYHRMLVHRVAAYFGLEHNVDS--------TGTS--VVVNKAKNTRVPENKF------------
XP_014675087.1             ----MSSYHRMLVHRVAAFFGLEHNIDS--------TGSC--VIVNKSKNTRMPEIKF------------
XP_030385838.1             ----ASSYNRMLIHRTAAFFGMDHNVDSE-------TQQC--VIVAVTKSTRIPEIRF------------
XP_029725418.1             ----MSSYNRMLIHRVAAYFGMEHNVDA--------TQQS--VIAEVTSATRIPEIRF------------
Dm_encore                  ----ASSYNRMLIHRTAAFFGMEHNVDTE-------TQQC--VIVAVAKNTRIPEIRF------------
XP_032599088.1             ----TSSYNRMLIHRTAAFFGMEHNVDSE-------TQQC--VIVAVSKGTRIPEIRF------------
KNC31935.1                 ----ASSYNRMLIHRTAAFFGMEHNVDTE-------TQQC--VIVAPTKNTRIPEIRF------------
XP_020714208.1             ----ASSYNRMLIHRTAAFFGMEHNVDTE-------TQQC--VIVAATKSTRIPEIRF------------
XP_023246188.1             ----MSSYNRMLVHRVAAYFGMEHNVDQ--------TGQC--VIVTRTKHMRIPDVRF------------
XP_023329389.1             ----MSSYHRMLVHRVAAYFGLDHNVDQ--------TGNC--VIVNKTKTTRLPDMKF------------
XP_029201356.1             ----MTSYHRMIVHRVAAYFGMDHNVDQQ-------TGKS--VIINKTMNTRIPESRF------------
XP_021368844.1             ----MSSYDRMLVHRIAAFFGLDHNVDQ--------TGKC--VIVNKTSNTRVPEFNF------------
XP_034305772.1             ----MSSYDRMLVHRIAAYFGLDHNVDQ--------TGKC--VIVNKTSNKRIPDFSF------------
XP_013815153.1             ----MTSYHRMLLHRVAAYFGLEHNVDQ--------SGKS--VIVNKTSNTRIPDQKF------------
XP_016861070_ARPP21        ----MSSYQRMLVHRVAAYFGLDHNVDQ--------TGKS--VIINKTSSTRIPEQRF------------
Hs/Xl/Dr                   ----MTSYHRMLLHRAAAYFGMDHNVDQ--------TGKA--VIINKTSNTRIPDQRF------------
Consensus aa:              .....sS@.RhhhH+lA.@@.hph.h.p........s.....l.l.+o..s+hP...h............
Consensus ss:                   hhhhhhhhhhhhhh  eeee            e  eeeee        hhh
```

```
                            98
Rbs1                        LQELQ---LNKKPEE------------------------------------CASSSESIEKSNNNRIFRI
Ca                          IKAIE---SSDNNEGGEFVKIVSVAHKYKSENTATTTNNNTNTDAPEQPNKPAPIKK-------LMVRKI
XP_010757305.1              MLQPP---NAIKTPP----------------------------------------PN-------APAMKI
RMJ22032.1                  ALHAA----NNGTPP----------------------------------------QA-------MPAMKI
QGA13340.1                  HLRPV---ESHNTPP----------------------------------------PN-------APAMKI
OQE15951.1                  ELHAA----TNSTPP----------------------------------------PA-------LPAMKI
XP_002585083.1              ALYAA---NAMKTPP----------------------------------------PV-------VPAMKI
PGH19146.1                  VLHAT---NVNKTPP----------------------------------------LA-------IPAMKI
XP_033404196.1              MLHPA----NTNTPP----------------------------------------PS-------APAMKI
KAF2399143.1                GFSTP---STVANTP---------------------------------------PPA-------VQARKI
KAF1939774.1                QMVDP---AKGTNTP---------------------------------------PAE-------LPARKI
ELQ33482.1                  SIAAA---NESASS--------------------------------------TPPPA-------MLPRKI
TQS37745.1                  SLSN----PLNSAN--------------------------------------TPPPA---------TMKI
SZF03220.1                  SLSN----PPASGN--------------------------------------TPPPT---------TMKI
KFZ15736.1                  SISN----PPTTGH--------------------------------------TPPPT-------VPAMKI
XP_016645040.1              SIAE----STSAAN--------------------------------------TPPPV-------VIPRKI
KFH40507.1                  HWDPA--PNSSNNS--------------------------------------TPPPA-------VLPKKI
CCE34041.1                  NMTPQ--PDVSSSS--------------------------------------TPPPA-------VLPMKI
CEJ80227.1                  LMVPE--MSASTSS--------------------------------------TPPPA-------VLPRKI
KAB5577609.1                GIIV----NASSNS--------------------------------------SPAPA-------ILPRKI
XP_014168719.1              SLVAV---NPPSSN--------------------------------------TPPPT-------IQPKKI
PSR74187.1                  SIAQ----SASTAS--------------------------------------TPPPP-------VMPRKI
VBB75180.1                  SIAA----NTPNSS--------------------------------------SPAPA-------VMPRKI
XP_009848197.1              TIAP----STSNTS--------------------------------------SPAPA-------VIPRKI
KIH88101.1                  SFVPV--NTPESTN--------------------------------------APPQI-------VKPRKI
XP_030994999.1              SIAAA---NETSSS--------------------------------------TPPPA-------LLPRKI
KXJ91167.1                  SIMETTVPEEPEAP--------------------------------------APPLA-------MLPRKI
KKA30932.1                  SIAAS-----NKSP--------------------------------------TPPPP-------MIPMKI
Td                          CDIVP---SKQAAH--------------------------------------GKNRSTTP-QSKPRQVKI
AJV06700.1                  LQELQ---LNKKPEE------------------------------------CISSSESIEKSNNNRIFRI
GCF00085.1                  SYGRT---DATATNT----------------------------------------NS-------NKRYRI
OVA05989.1                  ADIPI---SLPQEES----------------------------------------NS-------VVKVAI
GEU59184.1                  ADIPV---NIPTEDI---------------------------------------SSS-------VVKVAI
OWM87646.1                  ADIPV---NLPSEEK------------------------------------------------SAVKVFI
XP_008808576.1              SDVPA---KQTENEK----------------------------------------TE-------HFKIAI
MQL81330.1                  SDVPA---KQQECDK----------------------------------------SE-------QVKIVM
XP_020699935.1              SDISV---KQHENER----------------------------------------AG-------QIKIFV
MQM11394.1                  SGVPV---KKPEDEK----------------------------------------PG-------QIKIVI
KMZ62657.1                  SEVPA---KHLENCN----------------------------------------SE-------NFKLVL
PKA67145.1                  SDIPT---ILPKKEE----------------------------------------KG-------KVRIVT
XP_009386294.1              SEVPA---KVSDRDI----------------------------------------TE-------QVKITI
XP_020254643.1              SEIPA---KQTENKV----------------------------------------TE-------QLKIAI
XP_020254456.1              SDIPL---KQPETNK----------------------------------------PH-------KVKIAI
OAY67162.1                  SEIPC---KVNDTET----------------------------------------RE-------KFRIAI
KAF3787699.1                SQIPA---NLPENEK----------------------------------------AE-------HVKIVI
XP_023741289.1              SDIPI---KQSENEK----------------------------------------KD--------VKIVL
KAE9465814.1                SEIPV---KQLENDK----------------------------------------HE-------PIKIFM
KAE8697742.1                SEIPA---NHSENDK----------------------------------------SE-------QIKIAI
XP_006402961.1              SEIPT--AKQSENGK----------------------------------------FE-------HMKVSI
XP_010552904.1              SEIPA---KQPENDK----------------------------------------PE-------VMKIAI
XP_022966122.1              SQIPA---RQSVDDE----------------------------------------PG-------CVKIAI
GER51925.1                  SQVPA---KDSENNK----------------------------------------LD-------QIKIVI
XP_020114255.1              SEVSA---QQPEKEI----------------------------------------AE-------KIKIAI
ERM99095.1                  CEIPA---KMSETDK----------------------------------------PE-------HVKIAI
XP_006654401.1              SEVPS---KQTRTEH---------------------------------------EAA-------EKLKFV
Dip1                        QKFLA----SSRNDH---------------------------------------ETA-------EKLKFV
KAF3341400.1                SKILP-----KQSEG---------------------------------------EAS-------DKKKIV
XP_034191896.1              KEHIR---DDLILSE-------------------------------------------------EPRRSI
XP_024218457.1              RDFVR---DDLLLPE-------------------------------------------------EPRRSI
GFG30713.1                  RDHIR---EEFLFPE-------------------------------------------------EPRRSI
XP_015115923.1              KEHIR---DDLLLTE-------------------------------------------------EPRRSI
XP_029828169.1              RDQIR----DDLLSE-------------------------------------------------EPKKSI
XP_022254084.1              NDLIH----DELVPD-------------------------------------------------EPKKLI
XP_015903829.1              QDFIK----DELLPE-------------------------------------------------EPKKSI
XP_023222768.1              REQIH----DELVPD-------------------------------------------------EPKKSI
XP_002423078.1              GELIR---DDFRFSE-------------------------------------------------EPRRCI
XP_019865105.1              KEHIK---EDIVFAE-------------------------------------------------EPRRSI
XP_022901769.1              ESYIR---DDIGFTE-------------------------------------------------EPRRSI
XP_017778862.1              KHHIK---EDIVFSE-------------------------------------------------EPRRSI
VEN60483.1                  KELIR---EDIVFAN-----------------------------------------D-------EPRRSI
KAF2898948.1                KTHLK---DDFFGE--------------------------------------------------EPRRSI
XP_030766515.1              KEHIK----EIFNDE-------------------------------------------------PPRRSI
XP_018019452.1              REHFC---DEMTPLP----------------------------------------DD-------LPRRSV
XP_014672054.1              RQLIR---DDSLGD---------------------------------------------------EPKRL
XP_014675087.1              MQLIR---EDASLD---------------------------------------------------EPKRL
XP_030385838.1              KSLVR---VDHRDD---------------------------------------------------SRKSI
XP_029725418.1              KTLIN---ESFSEE---------------------------------------------------PRKSI
Dm_encore                   QSLV-------RDD---------------------------------------------------ARKSI
XP_032599088.1              KSLVR---VDHRED---------------------------------------------------ARKSI
KNC31935.1                  KTLVI----NNRDD---------------------------------------------------SRKSI
XP_020714208.1              KSLVR----DHRDD---------------------------------------------------TRKSI
XP_023246188.1              KEHIR---DDLLHFV------------------------------------VDNNGKSSDEASVARQKSI
XP_023329389.1              RDHVP---EDAVLST-----------------------------------------S-------EPRRLI
XP_029201356.1              LELIP---REESEDL----------------------------------------EG-------AVPRLI
XP_021368844.1              KEHVD---ARDSQK---------------------------------------------------KITGI
XP_034305772.1              KEHID---NLDSAP---------------------------------------------------KVKSI
XP_013815153.1              CEHIK---DEKSDD--------------------------------------------------FQKRYI
XP_016861070_ARPP21         CEHLK---DEKGEE--------------------------------------------------SQKRFI
Hs/Xl/Dr                    SEHIK---DEKSCE--------------------------------------------------FQQRFI
Consensus aa:               ..h.....p............................................................I
Consensus ss:               hhh                                                                 ee
```

```
                              129
Rbs1                          LKRKEV--------GNECD------YKIDGNTRTPNSNLTANSNK-----------DQKIEIDDKSSTDL
Ca                            INKPVS--------DPTPG------QAPG-TPESEVSDLTKELDESKLISEDTGSSTPISESSENTTNNI
XP_010757305.1                MRRSGQ--------TGGGMS----GAGSTAPSSSAPSKATSEAGADG-----NEEGAGSSLGATPAKDRA
RMJ22032.1                    MRRN----------EGERPP----TSGSTATS----SKAASEAGDGND-----GDRTGSSAGATPAKDRM
QGA13340.1                    MRRN----------EDRNVG----ENGTT--GSSGPSMPVSEAGGDGGN---EDDRGGSSAGATPAKDRS
OQE15951.1                    MRRT----------DGERPG----GEGSTAASSSVPSKATSEAGDSGV----DGDRGGSSSGATPAKDRL
XP_002585083.1                MRRAGQ--------QGTSFS----VGGSTAPSSSVPSKTASEVGAEGS----GEDGTESSTAATPAKERQ
PGH19146.1                    MRRVGQ--------PGDKAS----ASGSTAASSSAPSKATSETGGEGCND-DERNGAASSSGATPAKERT
XP_033404196.1                MRRLGQ--------PGEKN-----SAGSTAPSSAVPSKTASESGVDGAND-EDRNGGTSSTGATPSKDRA
KAF2399143.1                  MRRGGD--------TKGNS------GLSTASNSEVPSKATSENGGD--------SSNSDVGQNASGDAKS
KAF1939774.1                  MRR-----------DDGKS------GTNTGANSRNASKTTSEMGGS--------DGSNDGEGNKD---KA
ELQ33482.1                    MRRGEE--------GDSAP------------ASAGPSKPTSEDG--------------SDAKDKNANANQ
TQS37745.1                    MRRGGD-------------------------TGPSPSKAVSESG--------------SDEKEKGLSAKE
SZF03220.1                    MRRGGD-------------------------SGPSPSKALSESG--------------SDGKEKGLSAKE
KFZ15736.1                    MRRGGD-------------------------NGGSPSKPTSETG--------------SDGKDKSLSAKE
XP_016645040.1                MRREDS--------GDKAV------------SSAGPSKPTSEAG--------------SDAKEKAAQLKE
KFH40507.1                    MRRGQD--------GDAGV------------ASATPSKPTSEAG-------------SDKENKDKPQGNQ
CCE34041.1                    MRRGQN---------GDGL------------MSTNGSKPGSESG--------------SEAKDK-LAASQ
CEJ80227.1                    MRRGQE--------GDSGP------------TSTSPSKAASEAGS-----------ELKDAKEKTSASNP
KAB5577609.1                  MRRGED--------GEFGP------------GSGGPSKTTSEAG--------------SDGKDK-AGADR
XP_014168719.1                MRRGED--------TELSL-------------SAGPSKANSETG--------------SDSKDKGARAKE
PSR74187.1                    MRRGEN--------GDFG--------------SSGPSKPTSEDG--------------SDAKDK-AAARE
VBB75180.1                    MRRGED--------GEFGP------------PSAAPSKPTSEAG--------------SDGKDK-SVPRE
XP_009848197.1                MRRGED--------GEFAP------------SSANPSKPTSEAG--------------SDPKDKPAASKE
KIH88101.1                    MRRGEE--------FDQPA-------------STGPSKPTSEDG--------------SDLKEKAPPAKE
XP_030994999.1                MRRGED--------SELAA------------SSGGPSKPTSEDG--------------SDSKDKNAPTKE
KXJ91167.1                    MRRGED--------DGSGH------------ASNSASKATSENG--------------SDGTGK-PKTKE
KKA30932.1                    MRRGED--------GEPAM------------SSASQSKATSEVG--------------SEQKEKPVVKET
Td                            MKRVVD--------TPSSP----------------PSRL-------------------STPASQTVAEAK
AJV06700.1                    LKRKEV--------GNECD------YKIDGNTRTPNSNLTANSNK-----------DQKIEIDDKSSTDL
GCF00085.1                    LKRRDD--------QNNLD------KNGGNSNDNNDIIKFQEEQK-----------KDTRGNNDDTRVSL
OVA05989.1                    KQRPQK--------RSQMG------NSA---------------------------------NSHSSKSNH
GEU59184.1                    KQRPNK--------RSQG-------GGA---------------------------------NSNSLKNNS
OWM87646.1                    KQRPQK--------QSLHA------GGG---------------------------------NSKSLKSSG
XP_008808576.1                RQRPNK--------VSHSD-----------------------------------------LTELETKRNL
MQL81330.1                    RTKPNR--------TSSID-----------------------------------------ATETGIR-HT
XP_020699935.1                RRKPSK--------ASLCD-----------------------------------------TSEAG-NMSL
MQM11394.1                    RPRAVR--------ASSND-----------------------------------------VIGNEIKQVS
KMZ62657.1                    RQRPNI--------ASQND-----------------------------------------VDRPGIKNNP
PKA67145.1                    RPRASK--------TFRTG-----------------------------------------AVEPGAK-VI
XP_009386294.1                RPRPRM--------VSSGA-----------------------------------------GIDAVPEKSA
XP_020254643.1                RQRPSK--------TSLSD-----------------------------------------RTELENRKNS
XP_020254456.1                LPRQDK--------PSMAD-----------------------------------------SVHLGMKRNT
OAY67162.1                    RQRPNK--------VSQSD-----------------------------------------ANVLETKKNL
KAF3787699.1                  RPRPQK--------SYPSD-----------------------------------------ASDPNINTSP
XP_023741289.1                KPRPK---------SSFDG-----------------------------------------SNDQGKKCNL
KAE9465814.1                  RPRPSK--------ILSEQ-----------------------------------------ASGEGIKGNS
KAE8697742.1                  KPRPNN--------GSVNE-----------------------------------------ANQIGIKRSP
XP_006402961.1                KTRPSK--------GSGYG-----------------------------------------SGELEKKGGH
XP_010552904.1                KPRPTR--------GWGEG-----------------------------------------AGAGGVHQNV
XP_022966122.1                RPRLNK---------LSDD-----------------------------------------AGNGMARQCL
GER51925.1                    RPRPK---------ASSRD-----------------------------------------TSMLGLKDGQ
XP_020114255.1                RPRPRS--------ASSQN-----------------------------------------AAEL-YNKSG
ERM99095.1                    KPRPQK--------AAPND-----------------------------------------ATDQGIKRSA
XP_006654401.1                ICQRPR--------AFQNG-----------------------------------------AGDAGAKNGA
Dip1                          IYQRPK--------AFQNG-----------------------------------------SADSRNKSGV
KAF3341400.1                  LRQRPK--------ASTGD-----------------------------------------AMGSEAKRNL
XP_034191896.1                LKRDSS--------SFEDS------FNF-----------------------------KSPDRMSGDYCRQ
XP_024218457.1                LKRDSS--------SFDET------GSY-----------------------------KCGERMLGGDSRR
GFG30713.1                    LKRDSS--------SFEDG------CNFK--QSAQVLAVRDWQLRLDY-----ESLLQSPERQFSSESRR
XP_015115923.1                LKRDSN--------SFEDG------CFNF----------------------------KSPDRMSGDYCRR
XP_029828169.1                LKRDNA--------SFEDG-------------------------------------KESPERQSSTDSRR
XP_022254084.1                LKRDSV--------CFEDG-------------------------------------KESPERQSSVDSRK
XP_015903829.1                LKRDSI--------SFEEE------KER------------------------------SPERQSSTDSRR
XP_023222768.1                LKRDSA--------SFEEGKELSLPALLQ---------------------------QKSPERQLSTDGRH
XP_002423078.1                LKRDSS--------SFEEG------MQL-----------------------------RSPERQLSGDSRR
XP_019865105.1                LKRDSN--------SIEDY------GFK------------------------------SPDRSLSLENRR
XP_022901769.1                LKRDSN--------SMEDY------SFK------------------------------SPDRQSSYESRR
XP_017778862.1                LKRDSN--------SVEDY------NFK------------------------------SPERQSSMESKR
VEN60483.1                    LKRDSN--------SIEDY------------------------------------------RSCSSENRR
KAF2898948.1                  LKRDSS--------SIEDY------SFK------------------------------SPDRQCGLENRR
XP_030766515.1                LKRDSN--------SIEDY------CFK------------------------------SPERGYSLESRR
XP_018019452.1                LRREFS--------SFEDS------LGF------------------------------KQLADPLNDPRR
XP_014672054.1                LKREGAGSGGSFGSSFEDG------KGTH----------------------------SPGRQSSTESMRR
XP_014675087.1                LKRDNS--------SFEEP------RGMS----------------------------TPQRQSSTESMRR
XP_030385838.1                LKRDTH--------SFDEV------RQS-----------------------------TYLCPERFMLDRK
XP_029725418.1                LKRDTH--------SFDEY------RYG-----------------------------LLHCPDRGILDRK
Dm_encore                     LKRDTH--------SFDEV------RQS-----------------------------PYLCP--LSLDRK
XP_032599088.1                LKRDTH--------SFDEV------RQS-----------------------------PYLCPERVMLDRK
KNC31935.1                    LKRDTH--------SFDEA------RQSS----------------------------YLCPDRGGMLDRK
XP_020714208.1                LKRDTH--------SFDEA------RQSS----------------------------YLCPDRGSMLDRK
XP_023246188.1                LKRETN--------SFEDH------SHYK----------------------------SPDRLDDFCGNRR
XP_023329389.1                LKRESC--------SCDSG-----------------------------------------DRHSDNSSRR
XP_029201356.1                LKRQNT--------PSDDS------TPPK---------------------------IDSPVLIHSMEDKR
XP_021368844.1                MRRMGV-------GSLDDK------ESY-----------------------------CRSLDKPMLGSKK
XP_034305772.1                IQRENK--------SLDDR------DIY-----------------------------TRNLDKPSLAHTK
XP_013815153.1                LKRDNS--------SLDKD--------------------------------------DNQMRIRLKDDRR
XP_016861070_ARPP21           LKRDNS--------SIDKE------------------------------------DNQQNRMHPFRDDRR
Hs/Xl/Dr                      LKRDDA--------SMDRD--------------------------------------DNQIRVPLQDGRR
Consensus aa:                 bpR..p........s.....................................................p.
Consensus ss:                 e                                                                    hh
```

```
                              174
Rbs1                          E-----QERIEKERLYEQRKQEIFDKLN
Ca                            E-----SQRASKEALYMKLREEIFENNN
XP_010757305.1                T-----MSREEREAKYQEARERIFRDFP
RMJ22032.1                    I-----QTREEREAKYNEVRERIFRDFP
QGA13340.1                    T-----MTREEREAKYQEARERIFRDFP
OQE15951.1                    T-----LTREEREAKYQEARERIFRDFP
XP_002585083.1                A-----LTREEREAKYQEARERIFRDFP
PGH19146.1                    A-----LTREEREAKYQEARERIFRDFP
XP_033404196.1                N-----LTREEREAKYQEARERIFRDFP
KAF2399143.1                  K-----MTREEREAKYAEARQRIFGNVD
KAF1939774.1                  A-----LSREQREARYREARQRIFGSAE
ELQ33482.1                    K-----LTREEREEAYNKARLRIFGSSA
TQS37745.1                    KSIPLTLSREEREAAYNKARERIFGKEE
SZF03220.1                    K-----LSREEREAAYNKARERIFGKDE
KFZ15736.1                    K-----LSREEREAAYNKARERIFGNTE
XP_016645040.1                K-----MTQEEREEAYNRARERIFGSSE
KFH40507.1                    K-----LTREEREEVYRAARERIFGS--
CCE34041.1                    K-----LSREEREEMYKLARERIFGS--
CEJ80227.1                    K-----LSREQREEMYKLARERIFGN--
KAB5577609.1                  K-----LTREEREEKYNKARERIFGSVE
XP_014168719.1                T-----LSREAREEAYNKARERIFGSSM
PSR74187.1                    K-----MSREEREEAYNKARERIFGTTS
VBB75180.1                    K-----MTREEREEAYNKTRQRIFGSSE
XP_009848197.1                R-----MTREEREEAYNRARQRIFGNMK
KIH88101.1                    K-----MTREEREEAYNRARERIFAM--
XP_030994999.1                K-----LSREEREQKYNQARERIFGSAA
KXJ91167.1                    K-----LTREEREEAYKQARERIFGKED
KKA30932.1                    R-----KTREEREEAYNEARKRIFGSSN
Td                            E-----KDKLEKEERYKAARARIFQGVE
AJV06700.1                    E-----QERIEKERLYEQRKQEIFDKLN
GCF00085.1                    E-----RQRMEKEIQYEQRKQEIFSAPK
OVA05989.1                    A-----KSVEERKEEYNRARARIFSSNS
GEU59184.1                    L-----KSVEERKEEYNRARARIFSSS-
OWM87646.1                    A-----KSVEERKEEYNRARARIFNSNN
XP_008808576.1                A-----RTVEERKEEYDKARARIFSGSS
MQL81330.1                    L-----RTVEEREEEYDKARARIFSDSS
XP_020699935.1                M-----RTVEERMEEYDKARARIFSCSS
MQM11394.1                    S-----RTMEEREEDYDKARARIFSGCI
KMZ62657.1                    V-----RSVEERKEDYDKARARIFNNGS
PKA67145.1                    L-----RTVEERKEEYNKARARIFNDSF
XP_009386294.1                T-----RTVEERKEEYDRARARIFSGSC
XP_020254643.1                M-----RTVEERKEEYEKARARIFNS--
XP_020254456.1                M-----RTVEEREEEYDKARARIFNGSS
OAY67162.1                    L-----KTVEERKEEYDKARARIFSGTS
KAF3787699.1                  V-----RSVEERKEEYDKARARIFSSSS
XP_023741289.1                V-----RSVEERKEEYDRARARIFSTP-
KAE9465814.1                  A-----RTVEERKEEYDRARARIFSGSS
KAE8697742.1                  V-----RSVEERKEEYDRARARIFSSPS
XP_006402961.1                L-----RSVEERKEEYDRARARIFNDLT
XP_010552904.1                T-----RSVEERKEEYDKARARIFNSPT
XP_022966122.1                G-----RTVEQRMEEYDKARARIFSRHG
GER51925.1                    V-----RSVEERREEYDRARARIFNRPN
XP_020114255.1                L-----RTVEERKEEYDKARARIFSGSN
ERM99095.1                    V-----RSVEERKEEYDRARARIFNGSG
XP_006654401.1                A-----KTVEERIEEYNKARARIFNGS-
Dip1                          P-----KTVDERIEEYNKARARIFNGSI
KAF3341400.1                  M-----KSIEEREEEYDKARARIFSGSS
XP_034191896.1                N-----KSFEEREEEYERARRRIFKDS-
XP_024218457.1                S-----KSFEEREEEYEKIEGEIDCIDD
GFG30713.1                    S-----KSFEEREEEYEKARKRIFNRED
XP_015115923.1                S-----KSFEEREEEYEKVRRRIFKD--
XP_029828169.1                S-----KSIEEREEEYEKARARIFNQD-
XP_022254084.1                S-----KSIEEREEEYEKARARIFNQDS
XP_015903829.1                S-----KSFEEREEEYEKARARIFNQDE
XP_023222768.1                S-----KSFEEREEEYVKVRARIFKQDE
XP_002423078.1                S-----KSFEEREEEYERARKRIFNRDD
XP_019865105.1                S-----KSFEEREEEYEKVRRRIFNRE-
XP_022901769.1                S-----KSIEEREEEYKRARRRIFNNKE
XP_017778862.1                S-----KSFEEREEEYEKVRRRIFNKEM
VEN60483.1                    S-----KSFEEREEEYEKARRRIFNREH
KAF2898948.1                  S-----KSIEEREEEYERAKRRIFNSRE
XP_030766515.1                S-----KSFEEREEEYEKARRRIFRQEM
XP_018019452.1                S-----KSFEERQEEYHRARKRMFSQEV
XP_014672054.1                N-----KSIEEREEEYQKTRARIFNQD-
XP_014675087.1                T-----KSFEEREEAYEKTKARIYNHQD
XP_030385838.1                S-----KSFEEREEDFDRSRSRIFSRT-
XP_029725418.1                A-----KSFEEREEEYDKVKRRIFKNR-
Dm_encore                     A-----KSFEEREEDYDRARSRIFSRT-
XP_032599088.1                A-----KSFEEREEDYDRARSRIFNRTA
KNC31935.1                    A-----KSFEEREEDYDRARSRIFNRS-
XP_020714208.1                A-----KSFEEREEDYERARSRIFNRS-
XP_023246188.1                S-----KSFEEREAEYHMARKRIFRDEK
XP_023329389.1                S-----KSIEEREEDYERAKRRIFNDSS
XP_029201356.1                S-----KSIEEREEEYHKARERIFNQD-
XP_021368844.1                A-----QSFEERQQQYEKVRQKIFSSCD
XP_034305772.1                A-----RSFEERRKRYEKVRSKIFNSCD
XP_013815153.1                S-----KSIEEREEEYQRARERIFAQDS
XP_016861070_ARPP21           S-----KSIEEREEEYQRVRERIFAHDS
Hs/Xl/Dr                      S-----KSIEEREEEYQRVRERIFARES
Consensus aa:                 .......o.EERc..Ypch R.RIFs...
Consensus ss:                 h     hhhhhhhhhhhhhhhhhh
```

C

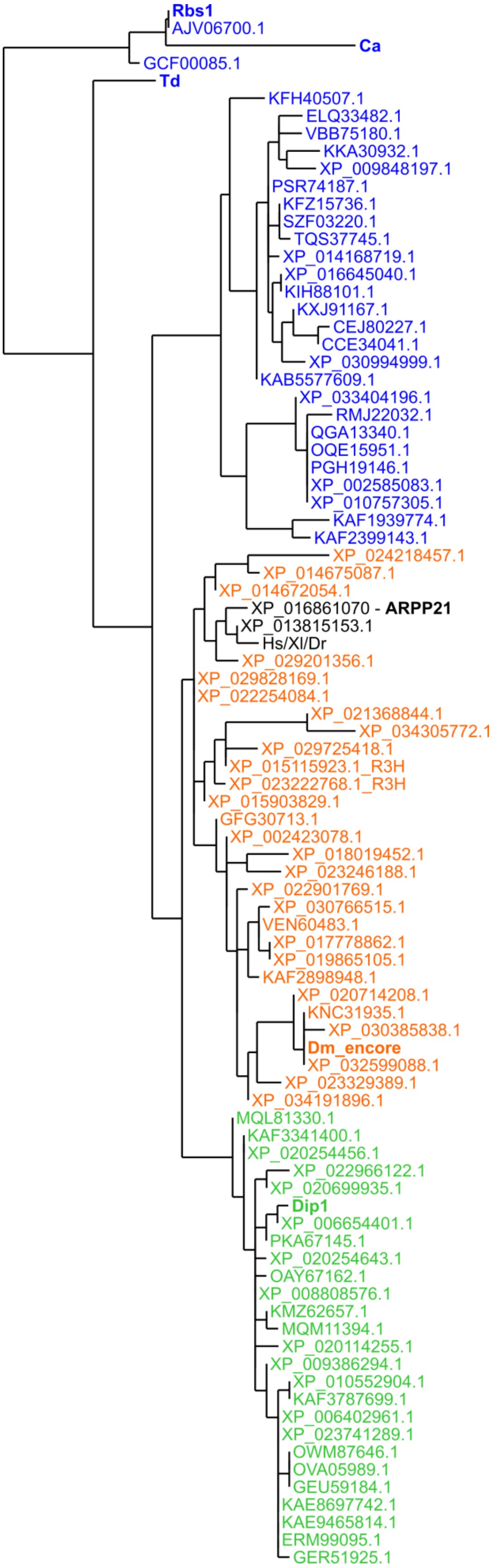
Rbs1
AJV06700.1
Ca
GCF00085.1
Td
KFH40507.1
ELQ33482.1
VBB75180.1
KKA30932.1
XP_009848197.1
PSR74187.1
KFZ15736.1
SZF03220.1
TQS37745.1
XP_014168719.1
XP_016645040.1
KIH88101.1
KXJ91167.1
CEJ80227.1
CCE34041.1
XP_030994999.1
KAB5577609.1
XP_033404196.1
RMJ22032.1
QGA13340.1
OQE15951.1
PGH19146.1
XP_002585083.1
XP_010757305.1
KAF1939774.1
KAF2399143.1
XP_024218457.1
XP_014675087.1
XP_014672054.1
XP_016861070 - ARPP21
XP_013815153.1
Hs/Xl/Dr
XP_029201356.1
XP_029828169.1
XP_022254084.1
XP_021368844.1
XP_034305772.1
XP_029725418.1
XP_015115923.1_R3H
XP_023222768.1_R3H
XP_015903829.1
GFG30713.1
XP_002423078.1
XP_018019452.1
XP_023246188.1
XP_022901769.1
XP_030766515.1
VEN60483.1
XP_017778862.1
XP_019865105.1
KAF2898948.1
XP_020714208.1
KNC31935.1
XP_030385838.1
Dm_encore
XP_032599088.1
XP_023329389.1
XP_034191896.1
MQL81330.1
KAF3341400.1
XP_020254456.1
XP_022966122.1
XP_020699935.1
Dip1
XP_006654401.1
PKA67145.1
XP_020254643.1
OAY67162.1
XP_008808576.1
KMZ62657.1
MQM11394.1
XP_020114255.1
XP_009386294.1
XP_010552904.1
KAF3787699.1
XP_006402961.1
XP_023741289.1
OWM87646.1
OVA05989.1
GEU59184.1
KAE8697742.1
KAE9465814.1
ERM99095.1
GER51925.1

Supplementary Figure S2 (seven previous pages). Sequences of proteins comprising R3H-SUZ domain combination. (**A**) Description of accession numbers of the retrieved sequences. The numbers are color-coded: blue for fungi, green for plants, orange for invertebrates and black for vertebrates. (**B**) Multiple sequence alignment obtained with Promals3D. Consensus secondary structure and sequence are given in the bottom of each segment. Amino acid number for Rbs1 are given on top of the alignment. For further explanation, see legend of Fig 10. (**C**) Phylogenetic tree of the analyzed sequences.
